# Cost-effectiveness of screening for chronic hepatitis B and C among migrant populations in a low endemic country

**DOI:** 10.1371/journal.pone.0207037

**Published:** 2018-11-08

**Authors:** Anita W. M. Suijkerbuijk, Albert Jan van Hoek, Jelle Koopsen, Robert A. de Man, Marie-Josee J. Mangen, Hester E. de Melker, Johan J. Polder, G. Ardine de Wit, Irene K. Veldhuijzen

**Affiliations:** 1 Centre for Nutrition, Prevention and Health Services, National Institute for Public Health and the Environment (RIVM), Bilthoven, the Netherlands; 2 Centre for Infectious Disease Control, National Institute for Public Health and the Environment (RIVM), Bilthoven, the Netherlands; 3 Department of Infectious Disease Epidemiology, Faculty of Epidemiology and Population Health, London School of Hygiene and Tropical Medicine, London, United Kingdom; 4 Department of Gastroenterology and Hepatology, Erasmus MC, Rotterdam, the Netherlands; 5 Tranzo Scientific Center for Care and Welfare, Tilburg School of Social and Behavioral Sciences, Tilburg University, Tilburg, The Netherlands; 6 Julius Centre for Health Sciences and Primary Health Care, University Medical Centre Utrecht, Utrecht, the Netherlands; Centre de Recherche en Cancerologie de Lyon, FRANCE

## Abstract

**Background:**

Chronic infection with hepatitis B or C virus (HBV/HCV) can progress to cirrhosis, liver cancer, and even death. In a low endemic country as the Netherlands, migrants are a key risk group and could benefit from early diagnosis and antiviral treatment. We assessed the cost-effectiveness of screening foreign-born migrants for chronic HBV and/or HCV using a societal perspective.

**Methods:**

The cost-effectiveness was evaluated using a Markov model. Estimates on prevalence, screening programme costs, participation and treatment uptake, transition probabilities, healthcare costs, productivity losses and utilities were derived from the literature. The cost per Quality Adjusted Life Year (QALY) gained was estimated and sensitivity analyses were performed.

**Results:**

For most migrant groups with an expected high number of chronically infected cases in the Netherlands combined screening is cost-effective, with incremental cost-effectiveness ratios (ICERs) ranging from €4,962/QALY gained for migrants originating from the Former Soviet Union and Vietnam to €9,375/QALY gained for Polish migrants. HBV and HCV screening proved to be cost-effective for migrants from countries with chronic HBV or HCV prevalence of ≥0.41% and ≥0.22%, with ICERs below the Dutch cost-effectiveness reference value of €20,000/QALY gained. Sensitivity analysis showed that treatment costs influenced the ICER for both infections.

**Conclusions:**

For most migrant populations in a low-endemic country offering combined HBV and HCV screening is cost-effective. Implementation of targeted HBV and HCV screening programmes to increase early diagnosis and treatment is important to reduce the burden of chronic hepatitis B and C among migrants.

## Introduction

People with chronic hepatitis B virus (HBV) and/or hepatitis C virus (HCV) infection are at risk of serious illness and death from liver disease, such as liver cirrhosis and hepatocellular carcinoma [1]. Transmission of HBV can occur vertically (mother-to-child), horizontally, and via sexual or blood contact, while HCV is mainly transmitted via blood contact [2]. The risk of developing chronic HBV infection is highly age dependent; 90% of infants infected at birth develop later in life a chronic infection, compared to less than 10% of those infected as adults [3]. Infection with HCV results in a chronic infection in 50–80% of patients [4]. Patients with chronic hepatitis B (CHB) or C (CHC) can eventually develop cirrhosis (up to 50%) and liver cancer (1–5%) over a period of 20–30 years [4, 5].

Effective antiviral treatment is available for CHB and can achieve long-term viral suppression in up to 94% of patients [5]. More recently, the treatment options for CHC have greatly improved through the introduction of direct acting antiviral therapy (DAAs), that shows cure rates of over 95% [6] and is, since November 2015, reimbursed by the basic healthcare insurance in the Netherlands for all CHC patients, independent of the stage of liver disease.

To prevent hepatitis-related burden of disease and death, timely diagnosis and linkage to care for treatment of eligible patients is needed [7]. However, this is challenging as many patients with chronic viral hepatitis experience no or few clinical symptoms before major complications (development of ascites, variceal bleeding, hepatocellular carcinoma (HCC) occur. Therefore, active case finding through screening is required.

The prevalence in the Dutch general population is low; estimated at 0.3% for chronic HBV and 0.1% for chronic HCV infections [8]. In a low endemic setting like the Netherlands, migrants originating from endemic regions are an important risk group and are estimated to account for 81% of chronic HBV and 60% of chronic HCV infections [9–11]. The Dutch Health Council advised in 2016 to offer screening for HBV and/or HCV to migrants from countries with a prevalence of chronic HBV or HCV of ≥2% [8]. To inform the implementation of targeted screening interventions we estimated the cost-effectiveness of screening foreign-born migrants for HBV, for HCV, and of combined HBV and HCV screening in the Netherlands.

## Methods

### Model

We investigated the cost-effectiveness of screening foreign-born migrants per country of origin using a Markov model programmed in MS Excel 2010. CHEERS guidelines for reporting economic evaluations were followed [12]. A lifelong time horizon was considered. The benefit of the screening programme is early detection of those who are prone to develop complications later in life, where antiviral treatment prevents disease progression, with associated quality of life advantages and cost reductions, as well as increased survival. The lifetime costs and Quality-Adjusted Life Years (QALYs) were calculated for a 40 year old person, based on the average age of migrants participating in several pilot screening projects performed in the Netherlands [13–16]. We present costs and clinical impact of HBV and HCV screening. The incremental cost-effectiveness ratio (ICER) of a HBV or HCV programme was calculated using no screening as comparator strategy. For a combined screening programme the ICER was first calculated for adding HCV testing to an HBV programme, and vice versa depending on the most cost-effective strategy. In addition, we performed country-specific threshold analysis to retrieve the maximum investment per migrant allowed, given the prerequisite that the cost-effectiveness level of €20,000 per QALY should not be exceeded. This value is considered an acceptable value for cost-effectiveness in the Netherlands [17]. The cost-effectiveness model was performed from a societal perspective, including an impact on productivity of those infected. Cost and QALYs were discounted differently, costs with 4% and health benefits with 1.5%, following Dutch guidelines [18].

### Hepatitis B

Disease states considered for HBV infection were undiagnosed inactive chronic infection, diagnosed inactive chronic infection, delayed clearance, CHB, compensated cirrhosis, decompensated cirrhosis, hepatocellular carcinoma (HCC), liver transplantation, hepatitis related death, and non-hepatitis related death (for a description of the disease states see S1 Table and S1 Fig). The annual transition rates for individuals experiencing natural disease progression and for those undergoing early treatment were taken from the literature and are shown in S2 Table. We assumed that all those with decompensated cirrhosis or HCC will seek medical care and have a known HBV status. The screening procedure and follow-up is described in supplementary S1 Text. In the initial screening test, 10% of those tested HBsAg positive were classified as CHB patients [19]. The remaining 90% had inactive chronic HBV infection.

### Hepatitis C

Disease states for HCV infection were CHC, compensated cirrhosis, decompensated cirrhosis, HCC, liver transplant, hepatitis-related death and non-hepatitis related death (see S1 Table and S2 Fig). The annual transition rates for individuals experiencing natural disease progression and for those undergoing treatment were taken from the literature and are given in S2 Table. Based on Helsper et al., it was assumed that 11% of CHC patients have compensated cirrhosis.[20] Patients with more severe disease end points, (i.e. decompensated cirrhosis and HCC), were assumed to seek medical care based on impaired health status, and got diagnosed and treated. The screening procedure and follow-up is described in S1 Text.

### Migrant population size and chronic HBV and HCV prevalence

Foreign-born migrant populations registered in the Municipal Personal Records database (BRP) in the Netherlands on 1-1-2016 (www.statline.nl) were included by country of birth for populations with at least 500 first generation migrants (aged 15 or over), see S3 and S4 Tables. In the Netherlands, asylum seekers and refugees are registered in the BRP within six months after arrival. As a consequence, unregistered foreign-born migrants and migrants coming from countries with less than 500 first generation migrants living in the Netherlands were excluded. The prevalence of chronic infections with HBV or HCV by country of birth was recently obtained from a review of the (un)published literature on prevalence in migrant populations in the Netherlands or, in absence of data, in countries of origin [11]. Migrant groups with an HBsAg prevalence estimate of at least 1% for HBV [21] and/or HCV-RNA prevalence of at least 0.5% for HCV in the country of origin were included [11, 22].

### Baseline testing rate and background mortality

In absence of a specific screening programme we assumed that 2.2% of the cohort migrants would be tested and diagnosed annually for HBV and 2.0% for HCV for other reasons such as pregnancy screening (HBV only), as part of STI testing or due to complaints. For HBV this was based on 900 newly diagnosed chronic HBV infections among foreign-born migrants in 2016 (notification data), of an estimated total of 40,000 chronic HBV infections in this group [11]. For HCV 500 chronic HCV diagnosis were reported by virological laboratories in 2016 of which ~60% were assumed to be foreign-born migrants (~300 cases) divided by an estimated 14,000 cases with chronic HCV infection among migrants [11, 23].

Background mortality for causes of death other than HBV and HCV disease was calculated using age-specific Dutch population averages retrieved from Statistics Netherlands [24]. Migrants, although born elsewhere, were assumed to have the Dutch background mortality rate.

### Participation in the screening programme

On the basis of experiences with earlier Dutch pilot screening programmes targeting migrants originating from Afghanistan, Iran, Iraq, the former Soviet Republics, Vietnam, China, and Egypt we assumed that 30% of all invited migrants would participate in the screening programme [14, 15, 25, 26]. Based on a Dutch retrospective analysis of follow-up diagnostics and referrals to secondary care after diagnosis of a hepatitis B or C infection in general practice and on data from screening interventions targeting Chinese migrants, we assumed that 80% of individuals that test positive would be effectively linked to care for clinical follow-up and treatment and the other 20% would experience natural disease progression [13, 27].

### Quality of life

Utility values to determine loss of quality of life in patients being chronically infected were derived from Stahmeijer et al [28] (Table 1). Given being in a certain health state, we assumed equal utility losses for HBV and HCV disease states, as both infections imply the same course of the disease except for having an inactive chronic infection, a health state only related to HBV.

**Table 1:** Overview of the costs of the screening programme, utilities and costs of HBV and HCV disease and treatment in Euro (2016).

	Hepatitis B	Source	Hepatitis C	Source
**Programme costs (in €) per person approached**				
	37	[29]	37	[29]
**Test costs (in €) per person screened**				
Order tariff	11	[30]	11	[30]
Test costs (HBsAg/anti-HCV)	10	[30]	10	[30]
Total test costs	21		21	
**Additional costs (in €) if positive**				
Outpatient visit	91	[18]	91	[18]
Order tariff	11	[30]	11	[30]
PCR	178	[30]	178	[30]
HBeAg	10	[30]	-	
ALT	2	[30]	-	
Fibroscan	103	[30]	103	[30]
Total additional costs	394		382	
**Annual healthcare costs (in €)**				
Inactive chronic infection	224	Own calculations^a^	-	-
CHB/ CHC	5386	Own calculations^a^	211	[31]
Compensated cirrhosis	6670	[32]	437	[31]
Decompensated cirrhosis	28,170	[31]	28,170	[31]
HCC	21,592	[31]	21,592	[31]
Liver transplantation	264,446	[32]^b^	264,446	[32]^b^
**Costs (in €) including treatment DAA once only**				
CHC	-	-	48,044	Own calculations^a^
Compensated cirrhosis	-	-	48,044	Own calculations^a^
Decompensated cirrhosis	-	-	48,044	Own calculations^a^
**Annual costs (in €) after treatment with DAAs**				
CHC	-	-	205	[31]
Compensated cirrhosis	-	-	501	[31]
Decompensated cirrhosis	-	-	501	[31]
**Productivity losses**				
*Annual number of work days lost*				
CHB/CHC (days)	8.4	[33]	13.2	[33]
Cirrhosis	15.6	[33]	25.2	[33]
HCC	18	[33]	27.6	[33]
Liver transplantation	26.4	[33]	38.4	[33]
*Employment rate*				
35–45 years	0.64	[34]	0.64	[34]
45–55 years	0.63	[34]	0.63	[34]
55–65 years	0.48	[34]	0.48	[34]
*Mean costs per working hour*				
40–44 years	40.04	[35]	40.04	[35]
45–49 years	41.20	[35]	41.20	[35]
50–54 years	41.61	[35]	41.61	[35]
55–59 years	41.83	[35]	41.83	[35]
60–64 years	41.30	[35]	41.30	[35]
**Utility values**				
Inactive chronic infection	1	assumption	-	
CHB/CHC	0.81	[28]	0.81	[28]
Compensated cirrhosis	0.74	[28]	0.74	[28]
Decompensated cirrhosis	0.72	[28]	0.72	[28]
HCC	0.72	[28]	0.72	[28]
Liver transplant	0.72	[28]	0.72	[28]
Post-liver transplant	0.79	[28]	0.79	[28]

^a^ see S5 Table for details

^b^ including 10 year follow-up costs

### Costs

Cost estimates were determined for medical care, productivity losses, and programme implementation and administration (Table 1 and S5 Table) and are explained in detail in S1 Text. Clinical management costs for inactive chronic HBV infection and for CHB patients were estimated using resource use based on clinical guidelines [5] and, following Dutch guidelines for economic evaluations in health care [18], multiplying resource use with Dutch references prices as presented in Table 1. We assumed similar healthcare costs for HCC and liver transplant for both HBV and HCV, as the clinical symptoms and treatment options are similar, irrespective of the original infection. Loss of productivity for CHB and CHC patients, up to 65 years of age was retrieved from Scalone et al. [33]. The sick leave length was multiplied by the average hourly wage adjusted for the employment rate in several age groups of migrants originating from non-western countries (Table 1) [18]. The costs of the screening programme included laboratory test costs as obtained from the Dutch Healthcare Authority [30], follow-up costs for an ultrasonography including fibroscan, and consulting clinicians based on reference prices from the National Health Care Institute [18]. Overall programme costs were estimated at €37 per person approached and included educating general practitioner (GPs), practice nurses, and Municipal Health Service (MHSs) staff, sending invitational letters to migrants, providing information in different languages on websites and in leaflets. All costs are indexed to Euros 2016

### Sensitivity and scenario analysis

To identify the most relevant uncertainties of our outcomes a one-way sensitivity analysis was performed. All input parameters in the model were decreased and increased with 25% and the ten most important ones are plotted in a tornado diagram. Furthermore, a number of scenario analyses were performed. For international comparison we applied a 3% discount rate for both costs and effects. Additionally, we changed screening participation from 30% to 20% and 40%, we changed the background mortality for the general population with the mortality for migrants originating from a non-western country [36], and we excluded productivity losses from the model. Finally, we assessed results for lower participation rates in combination with higher screening costs.

## Results

The clinical impact of screening ten migrant populations with the expected highest number of chronic HBV and ten migrant populations with the expected highest number of chronic HCV infections in the Netherlands is presented in Table 2, resulting in results for 16 countries as four countries had a relatively high number of both chronic HBV and HCV cases. These were Surinam, Vietnam, the former Soviet Union and Indonesia. The largest number of complications due to HBV-infection can be prevented by screening migrants born in Turkey, with an estimated number of 7,463 chronically infected cases in the Netherlands (S3 Table). The largest number of HCV complications can be averted by screening and treating migrants born in Surinam, with an estimated number of 2,935 chronically infected cases in the Netherlands (S4 Table).

**Table 2:** Averted cases of compensated cirrhosis, decompensated cirrhosis, HCC, liver transplant, and death over a life time period compared to no screening programme for the ten countries of origin with the expected highest number of infected HBV and HCV cases in the Netherlands^a^.

		Hepatitis B						Hepatitis C					
Country of origin	Infection with high number of cases	Chronic HBV	Comp. cirrhosis	Dec. cirrhosis	HCC	Liver transplant	Death	Chronic HCV	Comp. cirrhosis	Dec. cirrhosis	HCC	Liver transplant	Death
1. Turkey	HBV	739	333	91	262	80	412	11	4	2	2	0	2
2. Somalia	HBV	325	147	40	116	35	182	25	8	4	4	0	4
3. China	HBV	256	115	32	91	28	143	26	8	4	4	0	4
4. F. Yugoslavia	HBV	196	88	24	70	21	109	91	30	14	14	0	13
5. Surinam	HBV and HCV	178	80	22	63	19	99	597	195	91	89	0	85
6. Indonesia	HBV and HCV	156	70	19	55	17	89	106	35	16	16	0	15
7. F. Soviet Union	HBV and HCV	156	70	19	55	17	87	229	75	35	34	0	33
8. Vietnam	HBV and HCV	96	43	12	34	10	53	57	19	9	9	0	8
9. Cape Verde	HBV	94	43	12	34	10	53	30	10	5	4	0	4
10. Romania	HBV	94	43	12	33	10	53	76	25	12	11	0	11
11. Morocco	HCV	89	40	11	32	10	50	284	93	43	42	0	40
12. Syria	HCV	75	34	9	27	8	42	138	45	21	21	0	20
13. Poland	HCV	45	20	6	16	5	25	109	36	17	16	0	16
14. F. Dutch Antilles	HCV	19	9	2	7	2	11	97	32	15	14	0	14
15. Italy	HCV	63	28	8	22	7	35	87	28	13	13	0	12
16. Pakistan	HCV	31	14	4	11	3	17	77	25	12	12	0	11

Comp. = compensated, dec. = decompensated, HCC = hepatocellular carcinoma, F. = former, for Yugoslavia born before 1991, for Dutch Antilles born before 2010, and for the Soviet Union born before 1991

^a^ the number of countries does not sum up to 20 as the F. Soviet Union, Surinam, Vietnam, and Indonesia belong to the countries with the highest number of both HBV and HCV cases in the Netherlands

**Fig 1:**
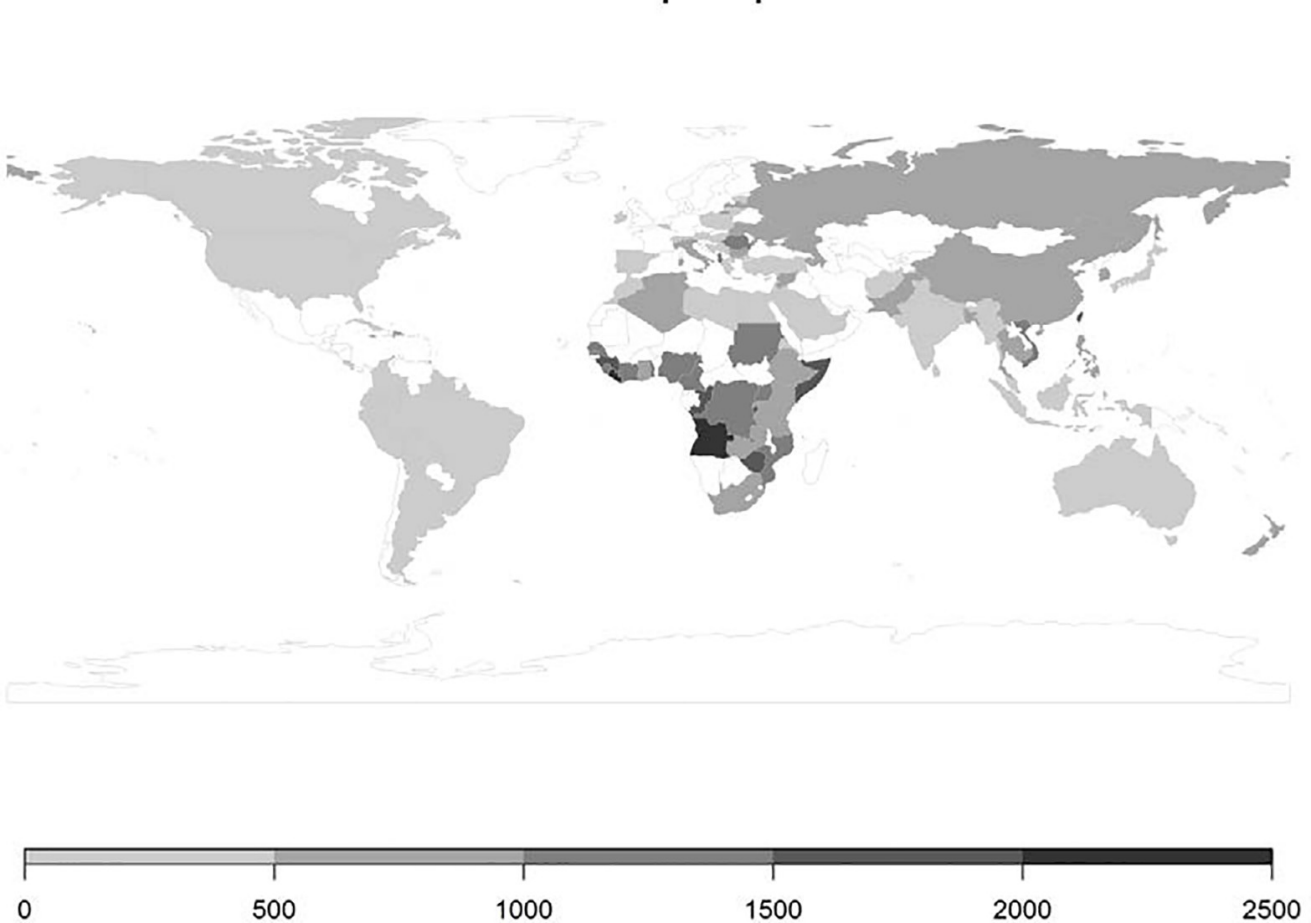
Maximum investment (€2016) allowed per migrant to achieve cost-effective combined HBV/HCV screening (results for migrants from the Former Soviet Union and born before 1991, Former Yugoslavia, born before 1991, and Former Dutch Antilles, born before 2010, are not included in this graph).

For most countries listed in Table 3 combining HBV and HCV screening is expected to be the most cost-effective strategy, with ICERs ranging from €4,962/QALY gained for migrants originating from the Former Soviet Union and Vietnam to €9,375/QALY gained for Poland. For those countries, solitary HBV or HCV screening is dominated as, compared to combined screening, for these strategies the costs per QALY gained are higher. For migrants originating from Turkey, screening only for HBV is the most cost-effective strategy. Adding HCV on top of HBV screening results in an ICER for HCV related costs and QALYs just above the Dutch threshold of €20,000 per QALY gained. HCV screening only is the most cost-effective option for migrants originating from Pakistan. However, extending HCV screening with HBV results in an ICER of around €5,000/ HBV related QALY gained, which is below the Dutch threshold. The ICER for combined screening was only marginally less beneficial compared to single HCV screening. The ICERs shown in Table 3 include default programme costs of €37 per person approached. Leaving these programme costs out of the model, the maximum investments allowed to arrive at an intervention which cost-effectiveness does not exceed the Dutch threshold of €20,000 per QALY gained can be calculated and is presented in Fig 1. More resource intensive strategies, i.e. higher program costs than €37 per participant, can be used for screening programmes targeting migrants from several African countries. For instance, combined screening of Somali migrants is still a cost-effective intervention at programme costs as high as €1,697 per migrant.

**Table 3:** Incremental cost-effectiveness of screening migrant groups for the ten countries of origin with the highest number of infected HBV and HCV cases in the Netherlands^a^.

Country		Δ Costs (€*1000)	Δ QALYs	ICER	Max. investment (€*1000)^b^	Max. investment / migrant (€)
1. Turkey	HBV	32,739	5,252	6,233	79,275	421
	HCV	8,408	72	dominated	-	-
	Both	34,174	5,324	dominated	79,270	421
2. Somalia	HBV	11,791	2,313	dominated	35,285	1,590
	HCV	1,538	154	dominated	2,369	107
	Both	12,508	2,467	5,070	37,654	1,697
3. China	HBV	10,488	1,816	dominated	27,537	601
	HCV	2,590	162	dominated	2,352	51
	both	11,381	1,979	5,752	29,889	652
4. F. Yugoslavia	HBV	8,682	1,394	dominated	21,050	423
	HCV	4,279	567	dominated	8,893	179
	both	11,119	1,961	5,670	29,943	601
5. Surinam	HBV	13,541	1,265	dominated	18,291	104
	HCV	21,601	3,722	dominated	59,367	337
	both	28,620	4,988	5,738	77,658	441
6. Indonesia	HBV	9,713	1,110	dominated	16,359	157
	HCV	6,995	661	dominated	10,082	96
	both	12,842	1,771	7,252	26,441	253
7. F. Soviet U	HBV	6,968	1,108	dominated	16,723	407
	HCV	7,149	1,430	dominated	22,970	559
	both	12,596	2,538	4,962	39,693	965
8. Vietnam	HBV	3,732	681	dominated	10,357	826
	HCV	1,882	357	dominated	5,716	456
	both	5,151	1,038	4,962	16,073	1,282
9. Cape Verde	HBV	3,646	671	dominated	10,205	876
	HCV	1,196	184	dominated	2,920	251
	both	4,410	855	5,157	13,124	1,126
10. Romania	HBV	3,869	670	dominated	10,155	600
	HCV	2,502	471	dominated	7,547	446
	both	5,744	1,141	5,034	17,702	1,045
11. Morocco	HBV	10,169	634	dominated	8,673	52
	HCV	13,855	1,771	dominated	27,729	166
	both	17,855	2,404	7,426	36,402	218
12.Syria	HBV	3,707	531	dominated	7,957	282
	HCV	4,442	857	dominated	13,751	487
	both	7,104	1,388	5,117	21,708	768
13. Poland	HBV	6,155	319	dominated	4,218	39
	HCV	7,225	682	dominated	10,413	96
	both	9,387	1001	9,375	14,631	136
14. F. Dutch Ant.	HBV	4,067	134	dominated	1,566	-
	HCV	5,705	604	dominated	9,312	117
	both	6,827	738	9,250	10,879	137
15. Italy	HBV	3,187	448	dominated	6,700	265
	HCV	3,129	543	dominated	8,656	343
	both	5,382	990	5,435	15,357	608
16. Pakistan	HBV	1,529	221	dominated	3,320	291
	HCV	2,306	483	4,778	7,769	682
	both	3,413	704	dominated	11,088	973

^a^ the number of countries does not sum up to 20 as the F. Soviet Union, Surinam, Vietnam, and Indonesia both belong to the countries with the highest number of HBV and HCV cases in the Netherlands

^b^ excluding €37 programme costs for HBV, HCV, or combined screening, F = former, for Yugoslavia born before 1991, for Dutch Antilles born before 2010, and for the Soviet Union born before 1991

Considering the Dutch reference value for cost-effectiveness, screening migrant groups for HBV has a cost-effectiveness level below €20,000/QALY at an HBV prevalence of 0.41%. HCV screening is cost-effective at a prevalence of 0.22%, taking the same threshold for cost-effectiveness into account. If we would perform combined screening of the total migrant population included in our study, i.e. those born in countries with a HBV prevalence of at least 1% and HCV prevalence of at least 0.5%, the ICER would be €5395 per QALY gained.

### Sensitivity and scenario analysis

**Fig 2:**
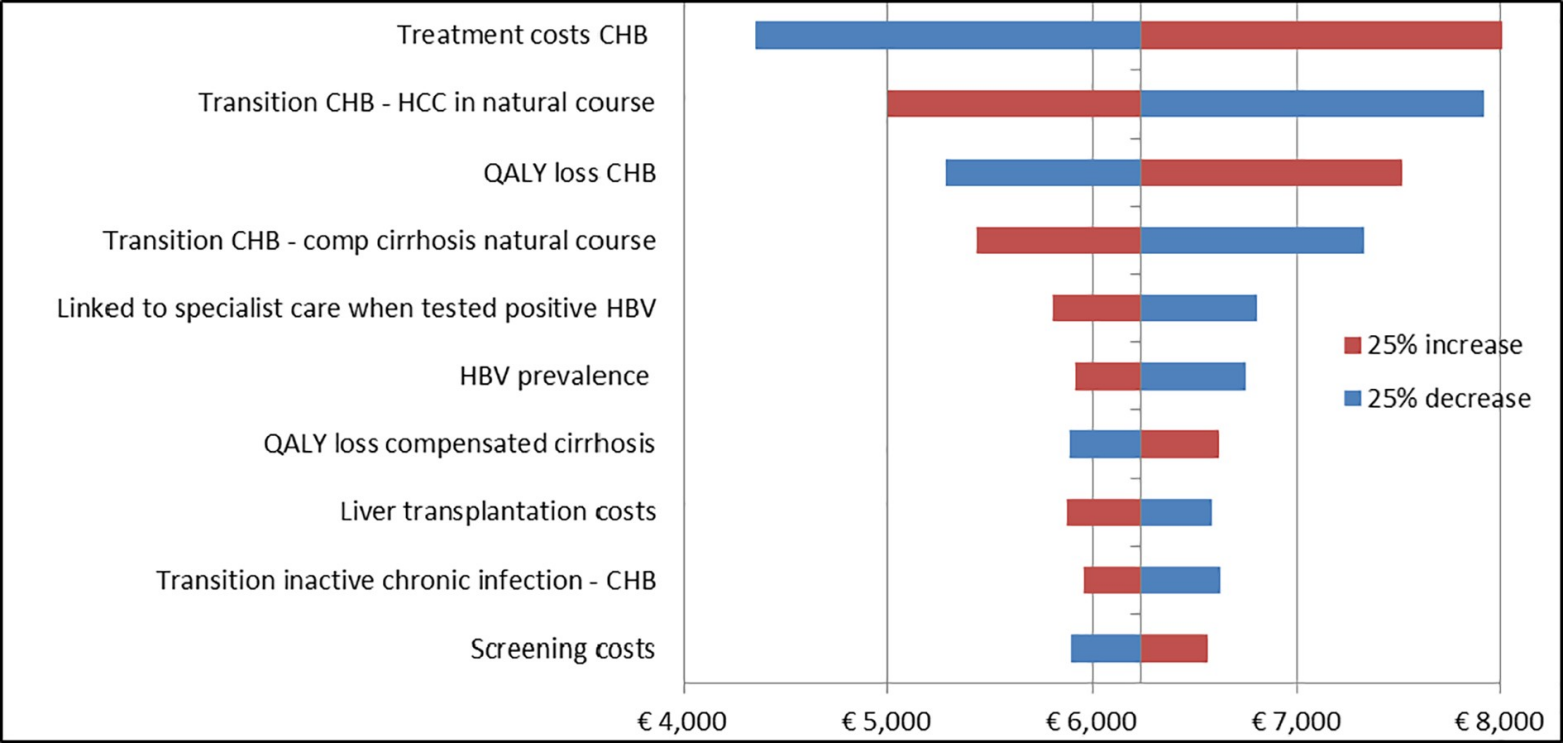
Sensitivity analysis for 10 most important HBV input parameters when decreasing and increasing them with 25% for Turkey, baseline ICER: €6233/QALY.

**Fig 3:**
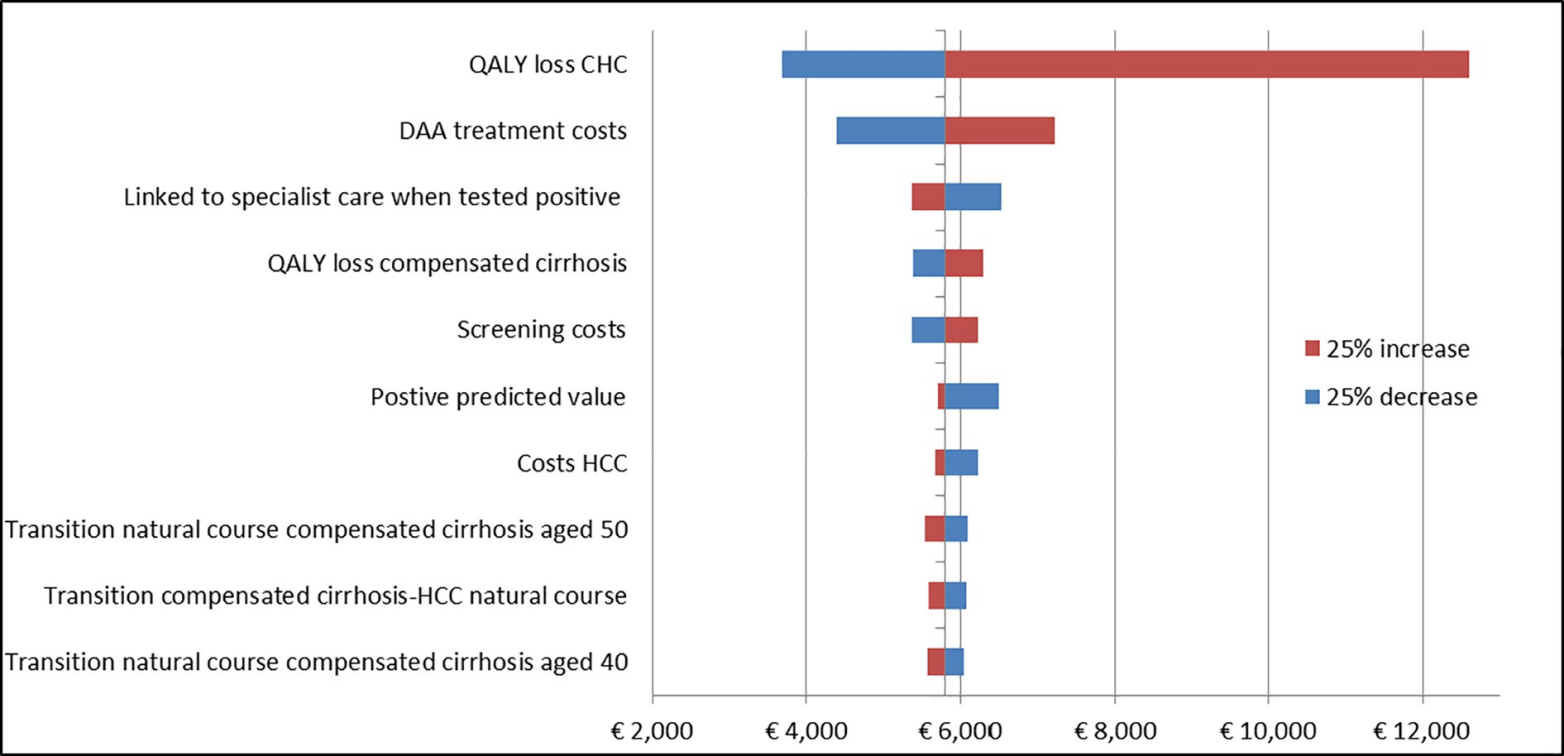
Sensitivity analysis for 10 most important HBV input parameters when decreasing and increasing them with 25% for Surinam, baseline ICER: €5803/QALY.

The ten most influential input parameters for HBV and HCV screening, after decreasing and increasing the input parameters with 25% are presented in Figs 2 and 3, using Turkey and Surinam as a high prevalence country for chronic HBV (4.0%) and HCV (1.7%) respectively. For both infections treatment costs highly influenced the ICER. With respect to HBV, the QALY loss for the CHB disease state and the transition probability from CHB to HCC and compensated cirrhosis were relatively important. For HCV, the QALY loss of CHC was important. In addition, discount rates for costs and QALYs were of significance for both infections, see supplementary S6 Table. If we excluded utility losses for the disease states CHB and CHC, that is assuming perfect health, the ICER increased to €7,519 for HBV and €12,605 for HCV. When the discount rate was changed to 3%, the ICER increased to respectively €10,426 and €6,838. Changing the background mortality and excluding productivity costs from the model, did not have much influence on both ICERs. If we decreased the participation rate to 10% and decreased the number of persons who will seek treatment after a positive test to 60%, the ICER increased to €10,346 for HBV and €11,187 for HCV.

## Discussion

Screening foreign-born migrants originating from HBV and HCV endemic countries with a seroprevalence of at least 0.41% for HBsAg and 0.22% for HCV-RNA is expected to be cost-effective in the Netherlands. These findings are driven by the prevention of long-term disease sequelae such as cirrhosis and HCC. For most migrant groups with an expected high number of CHB or CHC cases in the Netherlands this means that offering combined HBV and HCV screening is cost-effective with ICERs far below the reference value of €20,000 per QALY gained. We observed that screening for HBV or HCV alone was more cost-effective than combined screening for some migrant groups. For migrants from Turkey for instance, screening only for HBV was the most cost-effective option. This could be expected as the prevalence estimate for chronic HCV infection in Turkish migrants is 0.03%, which is well below our defined threshold for cost-effectiveness. For migrants from Pakistan screening for HCV only was more cost-effective than combined screening. However, the differences between the ICERS were very small and adding HBV on top of HCV was a cost-effective strategy as well. Therefore, and as the expected prevalence of both infections is over 2%, combined screening for Pakistani migrants is recommended.

Only few economic evaluations targeted at screening migrant populations have been published so far, however, none of these studies combined HBV and HCV screening. The cost-effectiveness of screening migrants in the UK for HCV was £23,200 per QALY gained [37]. However, in this study treatment with DAAs was not included yet. Screening migrants in the Netherlands for HBV was assessed at €8,966 per QALY gained which is in line with results from our study despite including the newly recommended treatment option of tenofovir and its associated costs [19]. The treatment options used in both models have comparable effectiveness, which might explain the similar outcomes. Screening refugees for HBV in the U.S.A. was cost-saving [38] while in Canada cost-effectiveness of screening refugees was estimated at $40,880 [39].

Not surprisingly, we found that reducing the currently high DAA treatment costs for CHC would reduce the ICER significantly. DAAs are highly effective in treating CHC, have a short duration of treatment, and are generally well tolerated even in patients with advanced liver disease [40] However, these advantages come with a major increase in treatment costs and financial consequences for health budgets [41]. In some countries, for example in India and Australia, generic DAAs are available at much lower prices [42–44]. Taking these lower prices into account, HCV screening could even be a cost-saving intervention according to Aggarwal et al.[42] However, in the Netherlands, the DAA patent period will last for several years and is unlikely to be violated, as long-term agreements on drug prices between the Ministry of Health and pharmaceutical companies have been made.

In this study, we adopted a societal perspective and included productivity losses in the model based on estimates taken from the literature [33]. However, Scalone et al assessed work days lost only for persons with a paid job while some persons lost their job due to disease. As a result productivity losses are probably underestimated in our study which results in a less beneficial cost-effectiveness.

As in any cost-effectiveness model, the final ICER is obtained by combining a selected set of parameter estimates. Therefore, there is uncertainty in the final ICER due to both statistical uncertainty in the parameter estimates, as well as due to our modelling decisions. We did not explore the contribution of the statistical uncertainty around each parameter on the ICER but we did explore which parameters are most influential in changing the ICER. This revealed that the ICER was mostly affected by treatment costs and utility losses of CHB and CHC. However, with regard to treatment costs, we included current market prices in baseline, and uncertainty relates primarily to the unknown lowest value of these market prices. Obviously, the ICER would only be affected positively with a further reduction in price of medication. If we excluded utility losses for CHB and CHC from the model, screening also remained cost-effective. Additionally, if we combined a low participation rate of only 10% with a relatively low rate of linkage to specialist care and treatment of 60%, screening migrant populations proved to be cost-effective. Given that even with extreme assumptions of the most influential parameters the ICER was still cost-effective we are confident that our conclusions are robust.

Results of this economic evaluation are largely driven by the underlying seroprevalence estimates of chronic HBV and HCV infection in the specific migrant groups. These seroprevalence figures have been retrieved from migrant screening projects and prevalence studies performed in the Netherlands and if unavailable, were taken from literature [21, 22, 45]. Due to several bias mechanisms, prevalences found in the Netherlands may be lower than in the country of origin. Migrants are not only a very heterogeneous group between countries, but also within countries, where migrant-groups from the same country can defer in socio-economic status, language and culture. We could not include undocumented migrants in our study, as numbers per country of birth are not available. However, as the total number of undocumented migrants in the Netherlands is estimated at 35.000 individuals, which is around 2% of the migrant population included in our study, we feel this would not have changed the results found [46].For all those reasons concerning the migrant populations, our cost-effectiveness estimates should be interpreted as an indication.

We were not able to estimate the proportion of migrants who have already been tested in the past. Therefore, we may have overestimated the number of people who can still benefit from screening. Furthermore, we used an average age of 40 years, based on the average age that was observed in several Dutch hepatitis screening projects. When the average person screened is older, the cost-effectiveness will be less beneficial as less QALYs can be gained. A younger age, in contrary, would result in a more cost-effective programme, presuming the same prevalence. But this latter is questionable, at least for HBV as vaccination started in some of these countries as early as in 2000 [7].

In our study we assumed a modest participation rate of 30% as baseline. Several interventions at low cost (see supplementary section 3) can improve engagement and compliance along the chronic viral hepatitis care continuum [47, 48]. As in most general practices only a few patients will be eligible for screening, a close collaboration between GPs, Municipal Health Services (MHS) and community-based organisations seems valuable in which MHSs can take responsibility for coordinating the screening programme [13, 14, 25, 26].

WHO launched a global viral hepatitis strategy aimed at reducing mortality by 65% by 2030. To reach this target, increased efforts are needed to scale-up testing and treatment [2]. Several countries have already made progress in recent years implementing interventions targeted at specific risk groups such as injecting drug users [49]. In addition, many countries have introduced universal HBV-vaccination and global coverage in infants has increased from around 30% in the year 2000 to over 80% in 2015. However, among migrants born before routine implementation of HBV-vaccination, the proportion of people living with chronic HBV-infection remains high [2]. Given that migrants are disproportionately affected by chronic viral hepatitis, both expansion of HBV and HCV screening programmes to increase early diagnose and improving access to effective treatment are important to reduce the burden of disease. Our results indicate cost-effectiveness of combined screening. Therefore, screening strategies can be considered targeting foreign-born migrants with a relatively high prevalence, not only for the Netherlands but also for other low endemic countries with a comparable healthcare system. The current advice of the Dutch Health Council is to screen migrants from countries with a prevalence of chronic HBV or HCV of ≥ 2%. Only 8.2% of all foreign-born migrants in the Netherlands meet this threshold, and this group includes 17.6% of all expected chronic HBV and HCV infections in the foreign-born population. When the prevalence threshold would be lowered to a prevalence of 1% for chronic HBV and 0.5% for chronic HCV, potentially 61.5% of all chronic infections could be detected by offering combined screening to 40% of the foreign-born migrant population. At an ICER of €5395 per QALY gained this strategy could reduce the HBV and HCV disease burden in a cost-effective way, and this suggests lowering of the recommended prevalence threshold for screening should be considered.

## Supporting information

S1 Text Screening procedure, costs, and design screening programme. (DOCX)

S1 Table HBV and HCV disease states. (DOCX)

S2 Table Annual transition rates. (DOCX)

S3 Table Population size and chronic HBV prevalence. (DOCX)

S4 Table Population size and chronic HCV prevalence. (DOCX)

S5 Table HBV and HCV treatment costs. (DOCX)

S6 Table Results sensitivity and scenario analysis. (DOCX)

S1 Fig Markov model for chronic HBV infection. (TIF)

S2 Fig Markov model for chronic HCV infection. (TIFF)
