# Supplementary material for: Cost-effectiveness of screening for chronic hepatitis B and C among migrant populations in a low endemic country
Source: PLoS One. 2018 Nov 8;13(11):e0207037. doi: 10.1371/journal.pone.0207037 (PMC6224111; doi:10.1371/journal.pone.0207037)
Supplement: S1 Text — (DOCX) [file pone.0207037.s001.docx]

**S1 Text**

1. **Screening procedure**

Migrants who participate in HBV screening will be tested for hepatitis B surface antigen (HBsAg). In case patients have a positive HBsAg test they are referred to specialist care for assessment of the severity of liver disease and eligibility for antiviral treatment according to clinical practice guidelines [1]. This assessment includes additional ultrasonography by fibroscan and laboratory tests such as HBeAg, HBV-DNA, and ALT levels [2]. Patients for whom antiviral therapy is currently not indicated are followed up by their GP and monitored yearly. Those with CHB are offered treatment with Tenofovir disoproxil fumerate (245 mg a day) and quarterly monitoring in specialist care [3]. The treatment was assumed to be 96% effective after four years of treatment [4].

Migrants who participate in HCV screening are tested for anti-HCV. In case of positive test results patients are referred to a clinician; an ultrasonography including fibroscan and an additional PCR are performed to confirm a chronic infection and assess the virus genotype [1]. Although several treatment combinations with direct acting antivirals (DAAs) exist [5], we assumed the regimen of Ledipasvir/Sofosbuvir (12 weeks) [6] to be the treatment of choice. This treatment is reimbursed in the Netherlands and 97% effective for most genotypes [7].

1. **Costs**

*Healthcare costs*

Clinical management costs for inactive chronic HBV infection and for CHB patients were calculated using assumed resource use based on clinical guidelines [8] and, following Dutch guidelines for economic evaluations [9], multiplying those with Dutch references prices as presented in Table 1. Treatment costs were obtained from an official Dutch site on drug prices (www.medicijnkosten.nl). CHC treatment costs for sofosbuvir/ledipasvir were taken from a Health Technology Assessment report of the National Healthcare Institute [10]. Other healthcare costs were based on a Dutch cost-effectiveness analysis of HBV vaccination for MSM [11] and an economic evaluation targeted at injecting drug users, also performed in the Netherlands [12]. We assume similar healthcare costs for HCC and liver transplant for both HBV and HCV as the clinical symptoms and treatment options are similar, irrespective of the original infection. For more details see Table S5.

*Productivity costs*

Loss of productivity for CHB and CHC patients, up to 65 years of age was retrieved from Scalone et al. [13]. In this study, all patients were on surveillance or treatment for chronic viral hepatitis, cirrhosis or HCC, or on post-treatment follow-up after liver transplantation. The sick leave length was multiplied by the average hourly wage adjusted for the employment rate in several age groups of migrants originating from non-western countries (Table 1) [9].

*Screening costs*

The costs of the screening programme included laboratory test costs obtained from the Dutch Healthcare Authority [14], follow-up costs for an ultrasonography including fibroscan, and consulting clinicians based on reference prices from the National Health Care Institute [9].

*Programme costs*

We did not have access to detailed cost figures of the different ways to implement screening programmes for migrants. Therefore, we took the average cost figure of costs of migrant screening from a study that describes different forms of migrant screening in Europe [15]. Overall programme costs were set at €37 per person approached and included educating general practitioner (GPs), practice nurses, and Municipal Health Service (MHSs) staff, sending invitational letters to migrants, providing information in different languages on websites and in leaflets based on mean costs of three combined HBV/HCV screening projects targeted at migrants: workplace-based outreach screening, opportunistic screening, and community outreach screening [15].

1. **Design of a screening programme**

Several examples of Dutch community based screening pilots exist to design a screening programme [16-18]. Moreover, other suggestions are presented in literature to improve the participation rate in a healthcare setting. GPs and practice nurses can offer general testing for HBV and HCV to migrants from medium- or high-prevalence countries who are registered at the general practice [19-22]. At first, registered foreign-born migrants can be invited for free testing by mail. Testing is also possible on an opportunistic, individual basis when a patient consults the GP or practice nurse for other health problems or when the patient is newly registered with the general practice. Language barriers in migrant groups at increased risk can be addressed by providing translated information or information in audio or visual formats, for example via leaflets and special websites. Posters and leaflet displays in the practice waiting rooms can help to raise awareness among minority groups at increased risk. GPs, practice nurses and other healthcare workers can attend an e-learning on testing and treating foreign-born migrants with HBV and HCV [23]. Finally, a screening programme can be integrated in a variety of healthcare facilities [24].

**References**

1. NHG-Werkgroep_Virushepatitis_en_andere_leveraandoeningen. NHG-Standaard Virushepatitis en andere leveraandoeningen (derde herziening). Huisarts Wet. 2016;59(3):108-19.

2. Buster EH, Baak BC, Bakker CM, Beuers UH, Brouwer JT, Drenth JP, et al. The 2012 revised Dutch national guidelines for the treatment of chronic hepatitis B virus infection. The Netherlands journal of medicine. 2012;70(8):381-5. Epub 2012/10/16. PubMed PMID: 23065990.

3. MDL. Richtlijn behandeling van chronische hepatitis-B-virusinfectie. Nederlandse Vereniging van Maag Darm Leverartsen; 2012.

4. Ridruejo E. Treatment of chronic hepatitis B in clinical practice with entecavir or tenofovir. World journal of gastroenterology. 2014;20(23):7169-80. Epub 2014/06/27. doi: 10.3748/wjg.v20.i23.7169. PubMed PMID: 24966587; PubMed Central PMCID: PMCPMC4064062.

5. EASL. EASL Recommendations on Treatment of Hepatitis C 2016. Journal of hepatology. 2017;66(1):153-94. Epub 2016/09/27. doi: 10.1016/j.jhep.2016.09.001. PubMed PMID: 27667367.

6. NIV/NVHB/NVMDL/NVH/NVZA. Richtsnoer behandeling hepatitis C. 2017.

7. Terrault NA, Zeuzem S, Di Bisceglie AM, Lim JK, Pockros PJ, Frazier LM, et al. Effectiveness of Ledipasvir-Sofosbuvir Combination in Patients With Hepatitis C Virus Infection and Factors Associated With Sustained Virologic Response. Gastroenterology. 2016;151(6):1131-40.e5. Epub 2016/10/25. doi: 10.1053/j.gastro.2016.08.004. PubMed PMID: 27565882; PubMed Central PMCID: PMCPMC5300778.

8. EASL. EASL 2017 Clinical Practice Guidelines on the management of hepatitis B virus infection. Journal of hepatology. 2017. Epub 2017/04/22. doi: 10.1016/j.jhep.2017.03.021. PubMed PMID: 28427875.

9. Zorginstituut_Nederland. Richtlijn voor het uitvoeren van economische evaluaties in de gezondheidszorg. Diemen: Zorginstituut Nederland, editor: ; 2015, 2015.

10. Reimbursement sofosbuvir, (2016).

11. Mangen MJ, Stibbe H, Urbanus A, Siedenburg EC, Waldhober Q, de Wit GA, et al. Targeted outreach hepatitis B vaccination program in high-risk adults: The fundamental challenge of the last mile. Vaccine. 2017;35(24):3215-21. Epub 2017/05/10. doi: 10.1016/j.vaccine.2017.04.068. PubMed PMID: 28483198.

12. van Santen DK, de Vos AS, Matser A, Willemse SB, Lindenburg K, Kretzschmar ME, et al. Cost-Effectiveness of Hepatitis C Treatment for People Who Inject Drugs and the Impact of the Type of Epidemic; Extrapolating from Amsterdam, the Netherlands. PloS one. 2016;11(10):e0163488. Epub 2016/10/07. doi: 10.1371/journal.pone.0163488. PubMed PMID: 27711200; PubMed Central PMCID: PMCPMC5053429.

13. Scalone L, Fagiuoli S, Ciampichini R, Gardini I, Bruno R, Pasulo L, et al. The societal burden of chronic liver diseases: results from the COME study. BMJ open gastroenterology. 2015;2(1):e000025. Epub 2015/10/16. doi: 10.1136/bmjgast-2014-000025. PubMed PMID: 26462277; PubMed Central PMCID: PMCPMC4599156.

14. NZA. Dutch Healthcare Authority. Available from: <www.nza.nl>.

15. Falla A, Veldhuijzen I, Rossi MK, Thomson R, Fernandez M, Cayla J, et al. Screening for chronic hepatitis B and C among migrants: outcomes and costs of different screening models. In: Ammon A, editor. ESCAIDE 2015; 11-13 November 2015; Stockholm. Stockholm2015. p. 69.

16. Richter C, Ter Beest G, Gisolf EH, P VANB, Waegemaekers C, Swanink C, et al. Screening for chronic hepatitis B and C in migrants from Afghanistan, Iran, Iraq, the former Soviet Republics, and Vietnam in the Arnhem region, The Netherlands. Epidemiology and infection. 2014;142(10):2140-6. Epub 2014/01/09. doi: 10.1017/s0950268813003415. PubMed PMID: 24398373.

17. Veldhuijzen IK, Wolter R, Rijckborst V, Mostert M, Voeten HA, Cheung Y, et al. Identification and treatment of chronic hepatitis B in Chinese migrants: results of a project offering on-site testing in Rotterdam, The Netherlands. Journal of hepatology. 2012;57(6):1171-6. Epub 2012/08/14. doi: 10.1016/j.jhep.2012.07.036. PubMed PMID: 22885717.

18. Zuure FR, Bouman J, Martens M, Vanhommerig JW, Urbanus AT, Davidovich U, et al. Screening for hepatitis B and C in first-generation Egyptian migrants living in the Netherlands. Liver international : official journal of the International Association for the Study of the Liver. 2013;33(5):727-38. Epub 2013/03/02. doi: 10.1111/liv.12131. PubMed PMID: 23448397.

19. NHS. Hepatitis B and C: ways to promote and offer testing to people at increased risk of infection. 2012 Contract No.: NICE public health guidance 43.

20. Heidrich B, Cetindere A, Beyaz M, Stahmeyer JT, Basaran MM, Braynis B, et al. High prevalence of hepatitis markers in immigrant populations: a prospective screening approach in a real-world setting. European journal of gastroenterology & hepatology. 2014;26(10):1090-7. Epub 2014/07/31. doi: 10.1097/meg.0000000000000164. PubMed PMID: 25076065.

21. Richmond JA, Sasadeusz J, Temple-Smith M. The Role of Primary Health Care in Hepatitis B Testing and Management: A Case Study. Journal of community health. 2017. Epub 2017/06/24. doi: 10.1007/s10900-017-0385-9. PubMed PMID: 28643212.

22. McLeod A, Cullen BL, Hutchinson SJ, Roy KM, Dillon JF, Stewart EA, et al. Limited impact of awareness-raising campaigns on hepatitis C testing practices among general practitioners. Journal of viral hepatitis. 2017. Epub 2017/05/16. doi: 10.1111/jvh.12724. PubMed PMID: 28502088.

23. Bechini A, Levi M, Falla A, Ahmad A, Veldhuijzen I, Tiscione E, et al. The role of the general practitioner in the screening and clinical management of chronic viral hepatitis in six EU countries. Journal of preventive medicine and hygiene. 2016;57(2):E51-60. Epub 2016/09/02. PubMed PMID: 27582629; PubMed Central PMCID: PMCPMC4996040.

24. Zuure FR, Urbanus AT, Langendam MW, Helsper CW, van den Berg CH, Davidovich U, et al. Outcomes of hepatitis C screening programs targeted at risk groups hidden in the general population: a systematic review. BMC public health. 2014;14:66. Epub 2014/01/24. doi: 10.1186/1471-2458-14-66. PubMed PMID: 24450797; PubMed Central PMCID: PMCPMC4016146.
