# Supplementary material for: Cost-effectiveness of screening for chronic hepatitis B and C among migrant populations in a low endemic country
Source: PLoS One. 2018 Nov 8;13(11):e0207037. doi: 10.1371/journal.pone.0207037 (PMC6224111; doi:10.1371/journal.pone.0207037)
Supplement: S1 Table — (DOCX) [file pone.0207037.s002.docx]

**S1 Table. HBV and HCV disease states**

| **Disease State** | **Description** |
| --- | --- |
| Delayed clearance | Spontaneous HBsAg loss resulting in persistent absence of HBsAg, the patient is no longer considered to have chronic infection; no viral treatment is indicated [1, 2]. We did not include this state in the HCV model. |
| Inactive chronic infection (HBV only) | Asymptomatic infection in which Hepatitis B virus is not actively replicating, with low ALT levels, no viral treatment is indicated [1-3]. |
| Chronic infection | The virus is actively replicating in the liver, but patients often experience no symptoms of infection [1, 2]. If aware of disease, antiviral treatment reduces liver damage and prevents appearance of further sequelae. In case of HCV infection the virus can be cleared through direct-acting antivirals [4]. |
| Compensated cirrhosis | Liver damage is present, but the patient often has no clinical symptoms [1, 2]. If aware of disease, antiviral treatment keeps infection under control and prevents further sequelae. In case of HCV infection the virus can be cleared through direct-acting antivirals [4]. |
| Decompensated cirrhosis | Significant scarring of the liver has occurred and the patient has severe and possibly life-threatening symptoms [1, 2]. Yearly treatment is indicated; sometimes a patient will undergo a liver transplant. In case of HCV infection the virus can be cleared through direct-acting antivirals [5] |
| Hepatocellular carcinoma | Cancer of the liver [1-3]. Yearly treatment is indicated; sometimes a patient will undergo a liver transplant. |
| Liver transplant | The liver of the patient with chronic infection is replaced with a donor liver by surgery [1, 2]. |
| HBV-related death/HCV-related death | Death from any cause related to chronic infection and sequelae. |
| Death from background causes | Death from any cause not related to chronic infection and sequelae. |

Note: transmission rates between the different HBV and HCV disease states are presented in S2 Table; Background mortality for causes of death other than HBV and HCV disease was calculated using age-specific Dutch population averages retrieved from Statistics Netherlands [39]

**References**

1. Fattovich G. Natural history of hepatitis B. Journal of hepatology. 2003;39 Suppl 1:S50-8. Epub 2004/01/08. PubMed PMID: 14708678.

2. Fattovich G, Bortolotti F, Donato F. Natural history of chronic hepatitis B: special emphasis on disease progression and prognostic factors. Journal of hepatology. 2008;48(2):335-52. Epub 2007/12/22. doi: 10.1016/j.jhep.2007.11.011. PubMed PMID: 18096267.

3. Schuppan D, Afdhal NH. Liver cirrhosis. Lancet (London, England). 2008;371(9615):838-51. Epub 2008/03/11. doi: 10.1016/s0140-6736(08)60383-9. PubMed PMID: 18328931; PubMed Central PMCID: PMCPMC2271178.

4. EASL. EASL Recommendations on Treatment of Hepatitis C 2016. Journal of hepatology. 2017;66(1):153-94. Epub 2016/09/27. doi: 10.1016/j.jhep.2016.09.001. PubMed PMID: 27667367.

5. Young J, Weis N, Hofer H, Irving W, Weiland O, Giostra E, et al. The effectiveness of daclatasvir based therapy in European patients with chronic hepatitis C and advanced liver disease. BMC infectious diseases. 2017;17(1):45. Epub 2017/01/08. doi: 10.1186/s12879-016-2106-x. PubMed PMID: 28061762; PubMed Central PMCID: PMCPMC5219681.
