# Supplementary material for: Cost-effectiveness of screening for chronic hepatitis B and C among migrant populations in a low endemic country
Source: PLoS One. 2018 Nov 8;13(11):e0207037. doi: 10.1371/journal.pone.0207037 (PMC6224111; doi:10.1371/journal.pone.0207037)
Supplement: S2 Table — (DOCX) [file pone.0207037.s003.docx]

**S2 Table. Overview of annual transition rates for progression following screening and natural progression of HBV and HCV**

|  | | **Natural course** | **Following screening** | **Source** |
| --- | --- | --- | --- | --- |
| **Hepatitis B** | |  |  |  |
| **From** | **To** |  |  |  |
| Inactive chronic HBV | Delayed clearance | 0.00425 |  | [1, 2] |
| infection | CHB | 0.02 |  | [2, 3] |
|  | HCC | 0.003 |  | [2, 3] |
| CHB | Inactive chronic infection | 0 | 0.3 | [1-5] |
|  | Delayed clearance | 0 | 0.008 | [1] |
|  | Compensated cirrhosis | 0.038 | 0.0045 | [1-3, 6] |
|  | HCC | 0.01 | 0.002 | [1-3, 6] |
|  | HBV-related death | 0.00002 | 0 | [5] |
| Compensated cirrhosis | Inactive chronic infection | 0 | 0.165 | [6] |
|  | Decompensated cirrhosis | 0.039 | 0.02 | [7-10] |
|  | HCC | 0.034 | 0.016 | [1-3, 6, 9] |
|  | HBV-related death | 0.049 | 0.024 | [3, 6, 9] |
| Decompensated cirrhosis | HCC | 0.06 | 0.06 | [2, 3, 9] |
|  | Liver transplant | 0.2 | 0.06 | [2, 3, 9] |
|  | HBV-related death | 0.26 | 0.26 | [1] |
| HCC | Liver transplant | 0.15 | 0.15 | [2, 3, 9] |
|  | HBV-related death | 0.35 | 0.35 | [2, 3, 9] |
| Liver transplant | HBV-related death | 0.064 | 0.064 | [11] |
| **Hepatitis C** | |  |  |  |
| **From** | **To** |  |  |  |
| CHC | Compensated cirrhosis (aged 40) | 0.009 | ^1^ | [12] |
|  | Compensated cirrhosis (aged 50) | 0.016 | ^1^ | [12] |
| Compensated cirrhosis | Decompensated cirrhosis | 0.044 | 0.003 | [13, 14] |
|  | HCC | 0.043 | 0.013 | [13, 15] |
| Decompensated cirrhosis | HCC | 0.035 | 0.035 | [15] |
|  | Liver transplant | 0.022 | 0.022 | [16] |
|  | HCV-related death | 0.28 | 0.13 | [15, 17] |
| HCC | Liver transplant | 0.017 | 0.017 | [18] |
| HCC | HCV-related death | 0.43 | 0.43 | [17] |
| Liver transplant | HCV-related death | 0.064 | 0.064 | [11] |

HCC=hepatocellular carcinoma, CHB=chronic hepatitis B infection, CHC=chronic hepatitis C infection, HBV=hepatitis B virus, HCV=hepatitis C virus, ^1^ after successful treatment, no disease progression is assumed

**References**

1. Fattovich G, Bortolotti F, Donato F. Natural history of chronic hepatitis B: special emphasis on disease progression and prognostic factors. Journal of hepatology. 2008;48(2):335-52. Epub 2007/12/22. doi: 10.1016/j.jhep.2007.11.011. PubMed PMID: 18096267.

2. Wong WW, Woo G, Jenny Heathcote E, Krahn M. Cost effectiveness of screening immigrants for hepatitis B. Liver international : official journal of the International Association for the Study of the Liver. 2011;31(8):1179-90. Epub 2011/07/13. doi: 10.1111/j.1478-3231.2011.02559.x. PubMed PMID: 21745300.

3. Hutton DW, Tan D, So SK, Brandeau ML. Cost-effectiveness of screening and vaccinating Asian and Pacific Islander adults for hepatitis B. Annals of internal medicine. 2007;147(7):460-9. Epub 2007/10/03. PubMed PMID: 17909207.

4. Wilkins T, Zimmerman D, Schade RR. Hepatitis B: diagnosis and treatment. American family physician. 2010;81(8):965-72. Epub 2010/04/15. PubMed PMID: 20387772.

5. Jazwa A, Coleman MS, Gazmararian J, Wingate LT, Maskery B, Mitchell T, et al. Cost-benefit comparison of two proposed overseas programs for reducing chronic Hepatitis B infection among refugees: is screening essential? Vaccine. 2015;33(11):1393-9. Epub 2015/01/18. doi: 10.1016/j.vaccine.2015.01.010. PubMed PMID: 25595868; PubMed Central PMCID: PMCPMC4633992.

6. Veldhuijzen IK, Toy M, Hahne SJ, De Wit GA, Schalm SW, de Man RA, et al. Screening and early treatment of migrants for chronic hepatitis B virus infection is cost-effective. Gastroenterology. 2010;138(2):522-30. Epub 2009/11/03. doi: 10.1053/j.gastro.2009.10.039. PubMed PMID: 19879275.

7. Fattovich G, Giustina G, Schalm SW, Hadziyannis S, Sanchez-Tapias J, Almasio P, et al. Occurrence of hepatocellular carcinoma and decompensation in western European patients with cirrhosis type B. The EUROHEP Study Group on Hepatitis B Virus and Cirrhosis. Hepatology (Baltimore, Md). 1995;21(1):77-82. Epub 1995/01/01. PubMed PMID: 7806171.

8. Fattovich G, Pantalena M, Zagni I, Realdi G, Schalm SW, Christensen E. Effect of hepatitis B and C virus infections on the natural history of compensated cirrhosis: a cohort study of 297 patients. The American journal of gastroenterology. 2002;97(11):2886-95. Epub 2002/11/12. doi: 10.1111/j.1572-0241.2002.07057.x. PubMed PMID: 12425564.

9. Kanwal F, Farid M, Martin P, Chen G, Gralnek IM, Dulai GS, et al. Treatment alternatives for hepatitis B cirrhosis: a cost-effectiveness analysis. The American journal of gastroenterology. 2006;101(9):2076-89. Epub 2006/09/14. doi: 10.1111/j.1572-0241.2006.00769.x. PubMed PMID: 16968510.

10. Realdi G, Fattovich G, Hadziyannis S, Schalm SW, Almasio P, Sanchez-Tapias J, et al. Survival and prognostic factors in 366 patients with compensated cirrhosis type B: a multicenter study. The Investigators of the European Concerted Action on Viral Hepatitis (EUROHEP). Journal of hepatology. 1994;21(4):656-66. Epub 1994/10/01. PubMed PMID: 7814813.

11. ELITA. Patient survival [26.2.2018]. Available from: <http://www.eltr.org/Specific-results-by-disease.html>.

12. Grishchenko M, Grieve RD, Sweeting MJ, De Angelis D, Thomson BJ, Ryder SD, et al. Cost-effectiveness of pegylated interferon and ribavirin for patients with chronic hepatitis C treated in routine clinical practice. International journal of technology assessment in health care. 2009;25(2):171-80. Epub 2009/04/01. doi: 10.1017/s0266462309090229. PubMed PMID: 19331708.

13. Cardoso AC, Moucari R, Figueiredo-Mendes C, Ripault MP, Giuily N, Castelnau C, et al. Impact of peginterferon and ribavirin therapy on hepatocellular carcinoma: incidence and survival in hepatitis C patients with advanced fibrosis. Journal of hepatology. 2010;52(5):652-7. Epub 2010/03/30. doi: 10.1016/j.jhep.2009.12.028. PubMed PMID: 20346533.

14. Coffin PO, Scott JD, Golden MR, Sullivan SD. Cost-effectiveness and population outcomes of general population screening for hepatitis C. Clinical infectious diseases : an official publication of the Infectious Diseases Society of America. 2012;54(9):1259-71. Epub 2012/03/14. doi: 10.1093/cid/cis011. PubMed PMID: 22412061; PubMed Central PMCID: PMCPMC3404694.

15. Townsend R, McEwan P, Kim R, Yuan Y. Structural frameworks and key model parameters in cost-effectiveness analyses for current and future treatments of chronic hepatitis C. Value in health : the journal of the International Society for Pharmacoeconomics and Outcomes Research. 2011;14(8):1068-77. Epub 2011/12/14. doi: 10.1016/j.jval.2011.06.006. PubMed PMID: 22152176.

16. Siebert U, Sroczynski G, Wasem J, Greiner W, Ravens-Sieberer U, Aidelsburger P, et al. Using competence network collaboration and decision-analytic modeling to assess the cost-effectiveness of interferon alpha-2b plus ribavirin as initial treatment of chronic hepatitis C in Germany. The European journal of health economics : HEPAC : health economics in prevention and care. 2005;6(2):112-23. Epub 2005/05/20. doi: 10.1007/s10198-005-0280-7. PubMed PMID: 15902546.

17. Fattovich G, Giustina G, Degos F, Tremolada F, Diodati G, Almasio P, et al. Morbidity and mortality in compensated cirrhosis type C: a retrospective follow-up study of 384 patients. Gastroenterology. 1997;112(2):463-72. Epub 1997/02/01. PubMed PMID: 9024300.

18. Razavi H, Elkhoury AC, Elbasha E, Estes C, Pasini K, Poynard T, et al. Chronic hepatitis C virus (HCV) disease burden and cost in the United States. Hepatology (Baltimore, Md). 2013;57(6):2164-70. Epub 2013/01/03. doi: 10.1002/hep.26218. PubMed PMID: 23280550; PubMed Central PMCID: PMCPMC3763475.
