# Supplementary material for: Cost-effectiveness of screening for chronic hepatitis B and C among migrant populations in a low endemic country
Source: PLoS One. 2018 Nov 8;13(11):e0207037. doi: 10.1371/journal.pone.0207037 (PMC6224111; doi:10.1371/journal.pone.0207037)
Supplement: S3 Table — (DOCX) [file pone.0207037.s004.docx]

**S3 Table. Population size and chronic HBV prevalence among foreign- born migrants in the Netherlands per country of birth (2016 01-01)**

| **Country** | **Population in NL** | **Point Prevalence (HBsAg)** | **Low Estimate** | **High estimate** | **Estimated average number of adults living with chronic HBV** | **Source** |
| --- | --- | --- | --- | --- | --- | --- |
| Afghanistan | 31998 | 2.67% | 0.94% | 4.40% | 855 | [1] |
| Albania | 1502 | 7.80% | 7.56% | 8.03% | 117 | [2] |
| Algeria | 3829 | 2.92% | 2.50% | 3.33% | 112 | [2] |
| Angola | 4803 | 12.49% | 11.09% | 13.88% | 600 | [2] |
| Argentina | 2946 | 0.77% | 0.77% | 0.78% | 23 | [2] |
| Armenia | 675 | 0.00% | 0.00% | 0.00% | - | [2] |
| Australia | 5112 | 0.37% | 0.36% | 0.38% | 19 | [2] |
| Austria | 5371 | 1.34% | 0.81% | 1.86% | 72 | [2] |
| Azerbaijan | 717 | 3.10% | 1.71% | 4.49% | 22 | [2] |
| Bangladesh | 933 | 3.10% | 2.99% | 3.21% | 29 | [2] |
| Bosnia and Herzegovina | 684 | 1.13% | 0.91% | 1.35% | 8 | [2] |
| Brazil | 13715 | 0.65% | 0.65% | 0.66% | 89 | [2] |
| Bulgaria | 19742 | 4.00% | 3.19% | 4.81% | 790 | [2] |
| Burundi | 2029 | 9.85% | 5.97% | 13.73% | 200 | [2] |
| Cambodia | 686 | 4.08% | 3.57% | 4.59% | 28 | [2] |
| Cameroon | 1639 | 12.25% | 11.71% | 12.78% | 201 | [2] |
| Canada | 5306 | 0.00% | 0.00% | 0.00% | - | [2] |
| Cape Verde | 11655 | 8.18% | 4.26% | 12.10% | 953 | [2] |
| China | 45842 | 5.63% | 4.25% | 7.00% | 2579 | [3] |
| Colombia | 8811 | 2.34% | 1.86% | 2.82% | 206 | [2] |
| Congo | 845 | 11.02% | 9.75% | 12.29% | 93 | [2] |
| Congo (Democratic Republic) | 4465 | 6.00% | 5.68% | 6.31% | 268 | [2] |
| Cuba | 1257 | 1.66% | 0.62% | 2.70% | 21 | [2] |
| Cyprus | 560 | 2.71% | 2.38% | 3.04% | 15 | [2] |
| Dominican Republic | 8291 | 4.45% | 2.65% | 6.25% | 369 | [2] |
| Ecuador | 2140 | 2.38% | 1.08% | 3.68% | 51 | [2] |
| Egypt | 11936 | 0.95% | 0.04% | 1.85% | 113 | [4] |
| Eritrea | 6492 | 2.50% | 2.32% | 2.67% | 162 | [2] |
| Estonia | 930 | 0.00% | 0.00% | 0.00% | - | [2] |
| Ethiopia | 10961 | 6.04% | 5.77% | 6.31% | 662 | [2] |
| Former Dutch Antilles | 79574 | 0.24% | 0.00% | 0.47% | 187 | [5] |
| Former Soviet Union | 41123 | 3.83% | 2.74% | 4.91% | 1573 | [6] |
| Former Yuguslavia | 49784 | 3.98% | 1.32% | 6.64% | 1981 | [7] |
| Gambia | 687 | 12.30% | 11.50% | 13.09% | 84 | [2] |
| Ghana | 13338 | 5.55% | 3.58% | 7.52% | 740 | [8] |
| Greece | 13240 | 0.97% | 0.95% | 1.00% | 128 | [2] |
| Guinea | 2311 | 15.09% | 14.16% | 16.01% | 349 | [2] |
| Guyana | 2245 | 1.32% | 0.72% | 1.91% | 30 | [7] |
| Hongkong | 9617 | 8.98% | 8.47% | 9.48% | 863 | [6] |
| India | 21848 | 1.46% | 1.44% | 1.47% | 318 | [2] |
| **Country** | **Population in NL** | **Point Prevalence (HBsAg)** | **Low Estimate** | **High estimate** | **Estimated average number of adults living with chronic HBV** | **Source** |
| Indonesia | 104480 | 1.51% | 0.21% | 2.81% | 1578 | [9] |
| Ireland | 4770 | 0.03% | 0.01% | 0.07% | 1 | [2] |
| Israel | 4743 | 0.96% | 0.93% | 0.99% | 46 | [2] |
| Italy | 25242 | 2.52% | 2.49% | 2.54% | 635 | [2] |
| Ivory Coast | 1049 | 9.42% | 8.70% | 10.14% | 99 | [2] |
| Jamaica | 932 | 3.97% | 2.65% | 5.29% | 37 | [2] |
| Japan | 5076 | 1.02% | 1.01% | 1.02% | 52 | [2] |
| Jordan | 856 | 1.87% | 1.68% | 2.06% | 16 | [2] |
| Kenya | 2046 | 5.17% | 4.86% | 5.48% | 106 | [2] |
| Kuwait | 1217 | 0.80% | 0.66% | 0.97% | 10 | [2] |
| Latvia | 3273 | 1.39% | 1.10% | 1.67% | 45 | [7] |
| Lebanon | 3057 | 1.22% | 1.10% | 1.34% | 37 | [10] |
| Liberia | 1575 | 17.63% | 15.70% | 19.55% | 278 | [10] |
| Libya | 1063 | 2.16% | 2.05% | 2.27% | 23 | [10] |
| Lithuania | 4563 | 1.71% | 1.55% | 1.86% | 78 | [10] |
| Luxemburg | 747 | 0.00% | 0.00% | 0.00% | - | - |
| Morocco | 166727 | 0.54% | 0.01% | 1.07% | 900 | [5] |
| Mozambique | 564 | 8.38% | 7.55% | 9.21% | 47 | [2] |
| Myanmar | 1061 | 3.40% | 3.26% | 3.54% | 36 | [2] |
| Nepal | 1475 | 0.82% | 0.80% | 0.84% | 12 | [2] |
| New Zealand | 1877 | 4.11% | 4.04% | 4.18% | 77 | [2] |
| Nigeria | 6016 | 9.76% | 9.59% | 9.93% | 587 | [2] |
| Pakistan | 11395 | 2.76% | 2.73% | 2.79% | 315 | [2] |
| Peru | 3503 | 2.11% | 1.90% | 2.32% | 74 | [2] |
| Philippines | 11737 | 4.63% | 4.53% | 4.73% | 543 | [2] |
| Poland | 107919 | 0.42% | 0.42% | 0.43% | 453 | [2] |
| Portugal | 15681 | 1.05% | 0.78% | 1.31% | 164 | [2] |
| Romania | 16936 | 5.62% | 5.50% | 5.73% | 951 | [2] |
| Russia | 2279 | 2.74% | 2.64% | 2.83% | 62 | [2] |
| Rwanda | 983 | 7.60% | 3.82% | 11.37% | 75 | [2] |
| Saudi Arabia | 1615 | 3.18% | 3.12% | 3.24% | 51 | [2] |
| Senegal | 894 | 11.06% | 10.72% | 11.40% | 99 | [2] |
| Serbia | 721 | 0.48% | 0.43% | 0.55% | 3 | [2] |
| Sierra-Leone | 3731 | 8.86% | 5.99% | 11.73% | 331 | [2] |
| Singapore | 2530 | 4.10% | 3.87% | 4.33% | 104 | [2] |
| Somalia | 22189 | 14.81% | 13.77% | 15.84% | 3285 | [2] |
| South Africa | 8857 | 6.70% | 6.56% | 6.83% | 593 | [2] |
| South Korea | 3497 | 4.37% | 4.36% | 4.37% | 153 | [2] |
| Spain | 21806 | 0.34% | 0.32% | 0.37% | 74 | [2] |
| Sri Lanka | 6755 | 2.61% | 1.90% | 3.31% | 176 | [2] |
| Sudan | 4342 | 9.79% | 9.03% | 10.54% | 425 | [2] |
| Surinam | 176284 | 1.02% | 0.51% | 1.53% | 1798 | [9] |
| Switzerland | 4660 | 0.18% | 0.10% | 0.33% | 8 | [2] |
| **Country** | **Population in NL** | **Point Prevalence (HBsAg)** | **Low Estimate** | **High estimate** | **Estimated average number of adults living with chronic HBV** | **Source** |
| Syria | 28254 | 2.67% | 2.17% | 3.17% | 754 | [2] |
| Taiwan | 2384 | 12.25% | 11.70% | 12.80% | 292 | [7] |
| Tanzania | 919 | 7.19% | 6.59% | 7.79% | 66 | [2] |
| Thailand | 12118 | 6.42% | 6.37% | 6.47% | 778 | [2] |
| Togo | 1108 | 11.52% | 7.45% | 15.59% | 128 | [2] |
| Trinidad and Tobago | 502 | 0.00% | 0.00% | 0.00% | - | - |
| Tunisia | 4317 | 6.18% | 5.95% | 6.40% | 267 | [2] |
| Turkey | 188450 | 3.96% | 2.62% | 5.30% | 7463 | [5] |
| Uganda | 1426 | 9.21% | 8.65% | 9.77% | 131 | [2] |
| Ukraine | 1169 | 1.50% | 1.10% | 1.89% | 17 | [2] |
| United Arab Emirates | 547 | 0.70% | 0.41% | 1.20% | 4 | [2] |
| Uruguay | 589 | 0.00% | 0.00% | 0.00% | - | [2] |
| USA | 20775 | 0.27% | 0.24% | 0.30% | 56 | [2] |
| Vietnam | 12539 | 7.72% | 5.86% | 9.58% | 968 | [1] |
| Zambia | 593 | 6.10% | 5.38% | 6.82% | 36 | [2] |
| Zimbabwe | 997 | 14.38% | 13.43% | 15.32% | 143 | [2] |

- = no data available

**References**

1. Richter C, Ter Beest G, Gisolf EH, P VANB, Waegemaekers C, Swanink C, et al. Screening for chronic hepatitis B and C in migrants from Afghanistan, Iran, Iraq, the former Soviet Republics, and Vietnam in the Arnhem region, The Netherlands. Epidemiology and infection. 2014;142(10):2140-6. Epub 2014/01/09. doi: 10.1017/s0950268813003415. PubMed PMID: 24398373.

2. Schweitzer A, Horn J, Mikolajczyk RT, Krause G, Ott JJ. Estimations of worldwide prevalence of chronic hepatitis B virus infection: a systematic review of data published between 1965 and 2013. Lancet (London, England). 2015;386(10003):1546-55. Epub 2015/08/02. doi: 10.1016/s0140-6736(15)61412-x. PubMed PMID: 26231459.

3. Coenen S, van Meer S, Vrolijk JM, Richter C, van Erpecum KJ, Mostert MC, et al. Clinical impact of five large-scale screening projects for chronic hepatitis B in Chinese migrants in the Netherlands. Liver international : official journal of the International Association for the Study of the Liver. 2016;36(10):1425-32. Epub 2016/03/24. doi: 10.1111/liv.13125. PubMed PMID: 27001619.

4. Zuure FR, Bouman J, Martens M, Vanhommerig JW, Urbanus AT, Davidovich U, et al. Screening for hepatitis B and C in first-generation Egyptian migrants living in the Netherlands. Liver international : official journal of the International Association for the Study of the Liver. 2013;33(5):727-38. Epub 2013/03/02. doi: 10.1111/liv.12131. PubMed PMID: 23448397.

5. Veldhuijzen IK, van Driel HF, Vos D, de Zwart O, van Doornum GJ, de Man RA, et al. Viral hepatitis in a multi-ethnic neighborhood in the Netherlands: results of a community-based study in a low prevalence country. International journal of infectious diseases : IJID : official publication of the International Society for Infectious Diseases. 2009;13(1):e9-e13. Epub 2008/08/06. doi: 10.1016/j.ijid.2008.05.1224. PubMed PMID: 18678518.

6. Kowdley KV, Wang CC, Welch S, Roberts H, Brosgart CL. Prevalence of chronic hepatitis B among foreign-born persons living in the United States by country of origin. Hepatology (Baltimore, Md). 2012;56(2):422-33. Epub 2011/11/23. doi: 10.1002/hep.24804. PubMed PMID: 22105832.

7. Grintjes K. Hepatitis C-detection among migrants in Nijmegen (HECOM). Tijdschrift voor Infectieziekten. 2014;9(5):126-33.

8. Zuure FR. Prevalence of serological markers of hepatitis B virus and hepatitis C virus infection among six ethnic groups living in Amsterdam, the Netherlands – The HELIUS study. Global Viral Hepatitis Summit – 15th International Symposium on Viral Hepatitis and Liver Disease (ISVHLD); Berlin2015.

9. Niessen WJ. Is inviting migrants for hepatitis B screening by letter post effective? Infectieziekten Bulletin. 2013;24(4):107-11.
