# Supplementary material for: Cost-effectiveness of screening for chronic hepatitis B and C among migrant populations in a low endemic country
Source: PLoS One. 2018 Nov 8;13(11):e0207037. doi: 10.1371/journal.pone.0207037 (PMC6224111; doi:10.1371/journal.pone.0207037)
Supplement: S4 Table — (DOCX) [file pone.0207037.s005.docx]

**S4 Table. Population size and chronic HCV prevalence among foreign- born migrants in the Netherlands per country of birth (2016 01-01)**

| **Country** | **population in NL** | **Point Prevalence (HCV-RNA)** | **Low Estimate** | **High estimate** | **Estimated average number of adults living with chronic HCV** | **Source** |
| --- | --- | --- | --- | --- | --- | --- |
| Afghanistan | 31998 | 0.65% | 0.00% | 1.29% | 206 | [1] |
| Albania | 1502 | 0.90% | 0.80% | 1.00% | 14 | [2] |
| Algeria | 3829 | 1.00% | 0.30% | 1.70% | 38 | [2] |
| Angola | 4803 | 3.50% | 0.10% | 6.90% | 168 | [2] |
| Argentina | 2946 | 0.75% | 0.30% | 1.20% | 22 | [2] |
| Armenia | 675 | 3.35% | 2.80% | 3.90% | 23 | [2] |
| Australia | 5112 | 0.85% | 0.70% | 1.00% | 43 | [2] |
| Austria | 5371 | 0.20% | 0.10% | 0.40% | 11 | [2] |
| Azerbaijan | 717 | 1.70% | 1.30% | 2.10% | 12 | [2] |
| Bangladesh | 933 | 0.95% | 0.20% | 1.70% | 9 | [3] |
| Bosnia and Herzegovina | 684 | 0.90% | 0.80% | 1.00% | 6 | [2] |
| Brazil | 13715 | 0.75% | 0.60% | 0.90% | 103 | [2] |
| Bulgaria | 19742 | 1.15% | 0.70% | 1.60% | 227 | [2] |
| Burundi | 2029 | 2.40% | 0.80% | 4.00% | 49 | [2] |
| Cambodia | 686 | 1.30% | 0.90% | 1.70% | 9 | [2] |
| Cameroon | 1639 | 0.65% | 0.50% | 0.80% | 11 | [2] |
| Canada | 5306 | 0.55% | 0.40% | 0.70% | 29 | [2] |
| Cape Verde | 11655 | 1.25% | 1.10% | 1.40% | 146 | [2] |
| China | 45842 | 0.28% | 0.06% | 0.50% | 128 | [4] |
| Colombia | 8811 | 0.75% | 0.60% | 0.90% | 66 | [2] |
| Congo | 845 | 3.50% | 0.10% | 6.90% | 30 | [2] |
| Congo (Democratic Republic) | 4465 | 3.50% | 0.10% | 6.90% | 156 | [2] |
| Cuba | 1257 | 0.30% | 0.10% | 0.70% | 4 | [2] |
| Cyprus | 560 | 0.00% | 0.00% | 0.00% | - | - |
| Dominican Republic | 8291 | 0.70% | 0.40% | 1.00% | 58 | [2] |
| Ecuador | 2140 | 0.45% | 0.30% | 0.60% | 10 | [2] |
| Egypt | 11936 | 1.66% | 0.43% | 2.89% | 198 | [5] |
| Eritrea | 6492 | 0.55% | 0.40% | 0.70% | 36 | [2] |
| Estonia | 930 | 1.25% | 0.90% | 1.60% | 12 | [2] |
| Ethiopia | 10961 | 0.55% | 0.40% | 0.70% | 60 | [2] |
| Former Dutch Antilles | 79574 | 0.60% | 0.40% | 0.80% | 477 | [2] |
| Former Soviet Union | 41123 | 2.75% | 2.10% | 3.40% | 1131 | [2] |
| Former Yuguslavia | 49784 | 0.90% | 0.80% | 1.00% | 448 | [2] |
| Gambia | 687 | 0.90% | 0.50% | 1.30% | 6 | [2] |
| Ghana | 13338 | 0.38% | 0.00% | 0.76% | 51 | [2] |
| Greece | 13240 | 1.10% | 0.70% | 1.50% | 146 | [2] |
| Guinea | 2311 | 1.25% | 1.10% | 1.40% | 29 | [2] |
| Guyana | 2245 | 0.60% | 0.40% | 0.80% | 13 | [2] |
| Hongkong | 9617 | 0.00% | 0.00% | 0.00% | - | - |
| India | 21848 | 0.60% | 0.40% | 0.80% | 131 | [2] |
| **Country** | **population in NL** | **Point Prevalence (HCV-RNA)** | **Low Estimate** | **High estimate** | **Estimated average number of adults living with chronic HCV** | **Source** |
| Indonesia | 104480 | 0.50% | 0.20% | 0.80% | 522 | [2] |
| Ireland | 4770 | 0.65% | 0.40% | 0.90% | 31 | [2] |
| Israel | 4743 | 1.00% | 0.70% | 1.30% | 47 | [2] |
| Italy | 25242 | 1.70% | 0.70% | 2.70% | 429 | [2] |
| Ivory Coast | 1049 | 1.25% | 1.10% | 1.40% | 13 | [2] |
| Jamaica | 932 | 0.60% | 0.40% | 0.80% | 6 | [2] |
| Japan | 5076 | 0.55% | 0.30% | 0.80% | 28 | [2] |
| Jordan | 856 | 0.30% | 0.10% | 0.40% | 3 | [2] |
| Kenia | 2046 | 0.20% | 0.10% | 0.30% | 4 | [2] |
| Kuwait | 1217 | 1.65% | 1.40% | 1.90% | 20 | [2] |
| Latvia | 3273 | 2.00% | 1.40% | 2.60% | 65 | [2] |
| Lebanon | 3057 | 0.20% | 0.10% | 0.40% | 6 | [2] |
| Liberia | 1575 | 1.25% | 1.10% | 1.40% | 20 | [2] |
| Libya | 1063 | 0.60% | 0.50% | 0.70% | 6 | [2] |
| Lithuania | 4563 | 1.00% | 0.70% | 1.30% | 46 | [2] |
| Luxembourg | 747 | 0.80% | 0.60% | 1.00% | 6 | [2] |
| Malaysia | 3017 | 1.05% | 0.80% | 1.30% | 32 | [2] |
| Morocco | 166727 | 0.84% | 0.00% | 1.68% | 1401 | [6] |
| Mozambique | 564 | 0.55% | 0.40% | 0.70% | 3 | [2] |
| Myanmar | 1061 | 0.65% | 0.50% | 0.80% | 7 | [2] |
| Nepal | 1475 | 1.00% | 0.70% | 1.30% | 15 | [2] |
| New Zealand | 1877 | 0.95% | 0.60% | 1.30% | 18 | [2] |
| Nigeria | 6016 | 1.20% | 1.00% | 1.40% | 72 | [2] |
| Pakistan | 11395 | 3.35% | 2.80% | 3.90% | 382 | [2] |
| Peru | 3503 | 0.45% | 0.30% | 0.60% | 16 | [2] |
| Philippines | 11737 | 0.45% | 0.30% | 0.60% | 53 | [2] |
| Poland | 107919 | 0.50% | 0.40% | 0.60% | 540 | [2] |
| Portugal | 15681 | 0.90% | 0.70% | 1.10% | 141 | [2] |
| Romania | 16936 | 2.20% | 1.80% | 2.60% | 373 | [2] |
| Russia | 2279 | 2.90% | 2.30% | 3.50% | 66 | [2] |
| Rwanda | 983 | 0.55% | 0.40% | 0.70% | 5 | [2] |
| Saudi Arabia | 1615 | 0.00% | 0.00% | 0.00% | - | - |
| Senegal | 894 | 1.25% | 1.10% | 1.40% | 11 | [2] |
| Serbia | 721 | 0.90% | 0.80% | 1.00% | 6 | [2] |
| Sierra-Leone | 3731 | 1.25% | 1.10% | 1.40% | 47 | [2] |
| Singapore | 2530 | 0.50% | 0.30% | 0.70% | 13 | [2] |
| Somalia | 22189 | 0.55% | 0.40% | 0.70% | 122 | [2] |
| South Africa | 8857 | 0.65% | 0.40% | 0.90% | 58 | [2] |
| South Korea | 3497 | 0.40% | 0.30% | 0.50% | 14 | [2] |
| Spain | 21806 | 0.75% | 0.30% | 1.20% | 164 | [2] |
| Sri Lanka | 6755 | 0.65% | 0.50% | 0.80% | 44 | [2] |
| Sudan | 4342 | 0.55% | 0.40% | 0.70% | 24 | [2] |
| Surinam | 176284 | 1.67% | 0.00% | 3.33% | 2935 | [6] |
| **Country** | **population in NL** | **Point Prevalence (HCV-RNA)** | **Low Estimate** | **High estimate** | **Estimated average number of adults living with chronic HCV** | **Source** |
| Switzerland | 4660 | 0.85% | 0.60% | 1.10% | 40 | [2] |
| Syria | 28254 | 2.40% | 1.30% | 3.50% | 678 | [2] |
| Taiwan | 2384 | 2.50% | 1.30% | 3.70% | 60 | [2] |
| Tanzania | 919 | 0.55% | 0.40% | 0.70% | 5 | [2] |
| Thailand | 12118 | 0.55% | 0.40% | 0.70% | 67 | [2] |
| Togo | 1108 | 1.25% | 1.10% | 1.40% | 14 | [2] |
| Trinidad and Tobago | 502 | 0.60% | 0.40% | 0.80% | 3 | [2] |
| Tunisia | 4317 | 0.65% | 0.20% | 1.10% | 28 | [2] |
| Turkey | 188450 | 0.03% | 0.00% | 0.06% | 57 | [6] |
| Uganda | 1426 | 0.55% | 0.40% | 0.70% | 8 | [2] |
| Ukraine | 1169 | 2.75% | 2.10% | 3.40% | 32 | [2] |
| United Arab Emirates | 547 | 1.05% | 0.50% | 1.60% | 6 | [2] |
| Uruguay | 589 | 0.60% | 0.30% | 0.90% | 4 | [2] |
| USA | 20775 | 0.95% | 0.70% | 1.20% | 197 | [2] |
| Vietnam | 12539 | 2.25% | 0.14% | 4.35% | 282 | [1] |
| Zambia | 593 | 0.55% | 0.40% | 0.70% | 3 | [2] |
| Zimbabwe | 997 | 0.65% | 0.40% | 0.90% | 6 | [2] |

- = no data available

**References**

1. Richter C, Ter Beest G, Gisolf EH, P VANB, Waegemaekers C, Swanink C, et al. Screening for chronic hepatitis B and C in migrants from Afghanistan, Iran, Iraq, the former Soviet Republics, and Vietnam in the Arnhem region, The Netherlands. Epidemiology and infection. 2014;142(10):2140-6. Epub 2014/01/09. doi: 10.1017/s0950268813003415. PubMed PMID: 24398373.

2. Polaris_Group. Global prevalence and genotype distribution of hepatitis C virus infection in 2015: a modelling study. The lancet Gastroenterology & hepatology. 2017;2(3):161-76. Epub 2017/04/14. doi: 10.1016/s2468-1253(16)30181-9. PubMed PMID: 28404132.

3. Gower E, Estes C, Blach S, Razavi-Shearer K, Razavi H. Global epidemiology and genotype distribution of the hepatitis C virus infection. Journal of hepatology. 2014;61(1 Suppl):S45-57. Epub 2014/08/03. doi: 10.1016/j.jhep.2014.07.027. PubMed PMID: 25086286.

4. hepatitisinfo.nl. Screening and awareness raising hepatitis B and hepatitis C among Chinese people in the Netherlands (2009-2013) 2017. Available from: <http://www.hepatitisinfo.nl/projecten/screening/DU2558_Screening-en-bewustzijnsbevorderinghepatitis-B-en-hepatitis-C-bij-Chinezen-in-Nederland-2009-2013.aspx>.

5. Zuure FR, Bouman J, Martens M, Vanhommerig JW, Urbanus AT, Davidovich U, et al. Screening for hepatitis B and C in first-generation Egyptian migrants living in the Netherlands. Liver international : official journal of the International Association for the Study of the Liver. 2013;33(5):727-38. Epub 2013/03/02. doi: 10.1111/liv.12131. PubMed PMID: 23448397.

6. Veldhuijzen IK, van Driel HF, Vos D, de Zwart O, van Doornum GJ, de Man RA, et al. Viral hepatitis in a multi-ethnic neighborhood in the Netherlands: results of a community-based study in a low prevalence country. International journal of infectious diseases : IJID : official publication of the International Society for Infectious Diseases. 2009;13(1):e9-e13. Epub 2008/08/06. doi: 10.1016/j.ijid.2008.05.1224. PubMed PMID: 18678518.
