# Supplementary material for: Cost-effectiveness of screening for chronic hepatitis B and C among migrant populations in a low endemic country
Source: PLoS One. 2018 Nov 8;13(11):e0207037. doi: 10.1371/journal.pone.0207037 (PMC6224111; doi:10.1371/journal.pone.0207037)
Supplement: S5 Table — (DOCX) [file pone.0207037.s006.docx]

**S5 Table. HBV and HCV treatment costs**

| **Hepatitis B** |  | **Euro 2016** | **Source/Notes** |
| --- | --- | --- | --- |
| Inactive chronic infection, annual costs | | € 224 | own calculations, annual follow-up costs at general practice (visit (n=1) and laboratory tests, ALAT, DNA amplification once a year) |
| Chronic HBV (CHB), annual costs | | € 5,386 | own calculations, annual costs (4 outpatient visits, laboratory tests (routine laboratory testing, serological tests, 4 times a year) and treatment with tenofovir) |
| Compensated cirrhosis, annual costs | | € 6,670 | Mangen [1] |
| Decompensated cirrhosis, annual costs | | € 28,170 | van Santen [2] |
| HCC, annual costs | | € 21,592 | van Santen [2] |
| Liver transplantation | | € 264,446 | Mangen, costs liver transplantation including 10 year follow-up costs [1] |
| **Hepatitis C** |  |  |  |
| Chronic HCV (CHC), annual costs | | € 211 | van Santen [2] |
| Compensated cirrhosis, annual costs | | € 437 | van Santen [2] |
| **Costs including treatment** | | | |
| Chronic HCV (CHC), Compensated cirrhosis,  Decompensated cirrhosis | | € 48,044 | once only costs based on 12-weeks treatment with ledipasvir/sofosbuvir including outpatient visits (n=7), routine laboratory testing and additional diagnostics (radiology, ECG) |
| HCC, annual costs | | € 21,592 | van Santen [2] |
| Liver transplantation | | € 264,446 | Mangen, costs liver transplantation including 10 year follow-up costs [1] |
| **Monitoring costs after successful treatment** | | |  |
| Chronic HCV, annual costs | | € 205 | van Santen [2] |
| Compensated cirrhosis, annual costs | | € 501 | van Santen [2] |
| Decompensated cirrhosis, annual costs | | € 501 | van Santen [2] |

**References**

1. Mangen MJ, Stibbe H, Urbanus A, Siedenburg EC, Waldhober Q, de Wit GA, et al. Targeted outreach hepatitis B vaccination program in high-risk adults: The fundamental challenge of the last mile. Vaccine. 2017;35(24):3215-21. Epub 2017/05/10. doi: 10.1016/j.vaccine.2017.04.068. PubMed PMID: 28483198.

2. van Santen DK, de Vos AS, Matser A, Willemse SB, Lindenburg K, Kretzschmar ME, et al. Cost-Effectiveness of Hepatitis C Treatment for People Who Inject Drugs and the Impact of the Type of Epidemic; Extrapolating from Amsterdam, the Netherlands. PloS one. 2016;11(10):e0163488. Epub 2016/10/07. doi: 10.1371/journal.pone.0163488. PubMed PMID: 27711200; PubMed Central PMCID: PMCPMC5053429.
