# Supplementary material for: Cost-effectiveness of screening for chronic hepatitis B and C among migrant populations in a low endemic country
Source: PLoS One. 2018 Nov 8;13(11):e0207037. doi: 10.1371/journal.pone.0207037 (PMC6224111; doi:10.1371/journal.pone.0207037)
Supplement: S6 Table — (DOCX) [file pone.0207037.s007.docx]

**S6 Table. Results sensitivity and scenario analysis**

|  | **Hepatitis B** | | **Hepatitis C** | |
| --- | --- | --- | --- | --- |
|  | **ICER**  **(costs €/QALY)** | **Relative change compared to main analysis (%)** | **ICER**  **(costs €/QALY)** | **Relative change compared to main analysis (%)** |
| **Main analysis** | 6,233 |  | 5,803 |  |
| **Discount rate 3%** | 10,426 | 67 | 6,838 | 18 |
| **Participation 20%** | 6,897 | 11 | 6,679 | 15 |
| **Participation 40%** | 5,902 | -5 | 5,365 | -8 |
| **Corrected background mortality** | 5,801 | -7 | 5,619 | -3 |
| **Without including productivity losses** | 6,404 | 3 | 6,527 | 12 |
| **Without including utility losses for a chronic HBV/HCV disease state** | 7,519 | 21 | 12,605 | 117 |
| **50% increase of screening costs and 60% of persons will seek treatment when tested positive** | 7,691 | 23% | 7,682 | 32% |
| **Participation 10% and 60% of persons will seek treatment when tested positive** | 10,346 | 66% | 11,187 | 93% |
