# Supplementary figures and images for: Cost-effectiveness of screening for chronic hepatitis B and C among migrant populations in a low endemic country
Source: PLoS One. 2018 Nov 8;13(11):e0207037. doi: 10.1371/journal.pone.0207037 (PMC6224111; doi:10.1371/journal.pone.0207037)

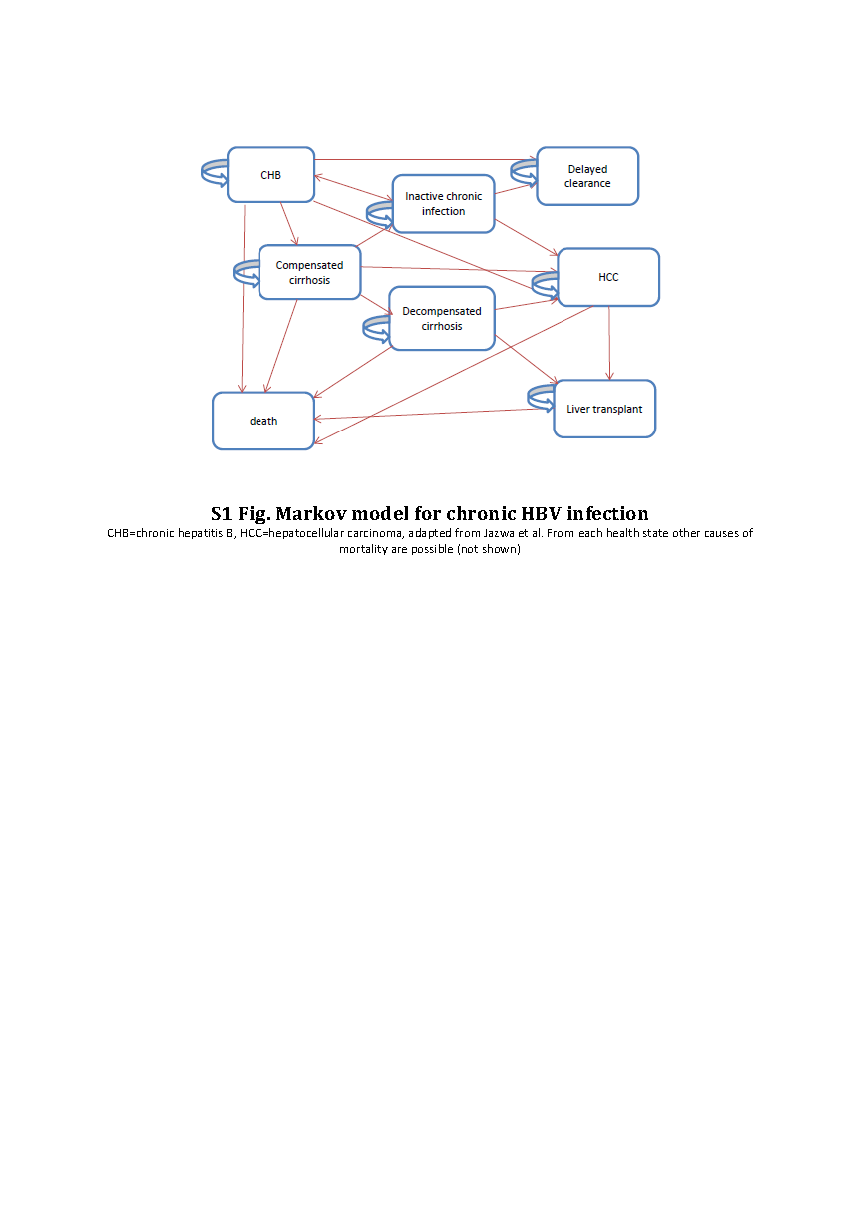

Supplement: S1 Fig — (TIF) [file pone.0207037.s008.tif]

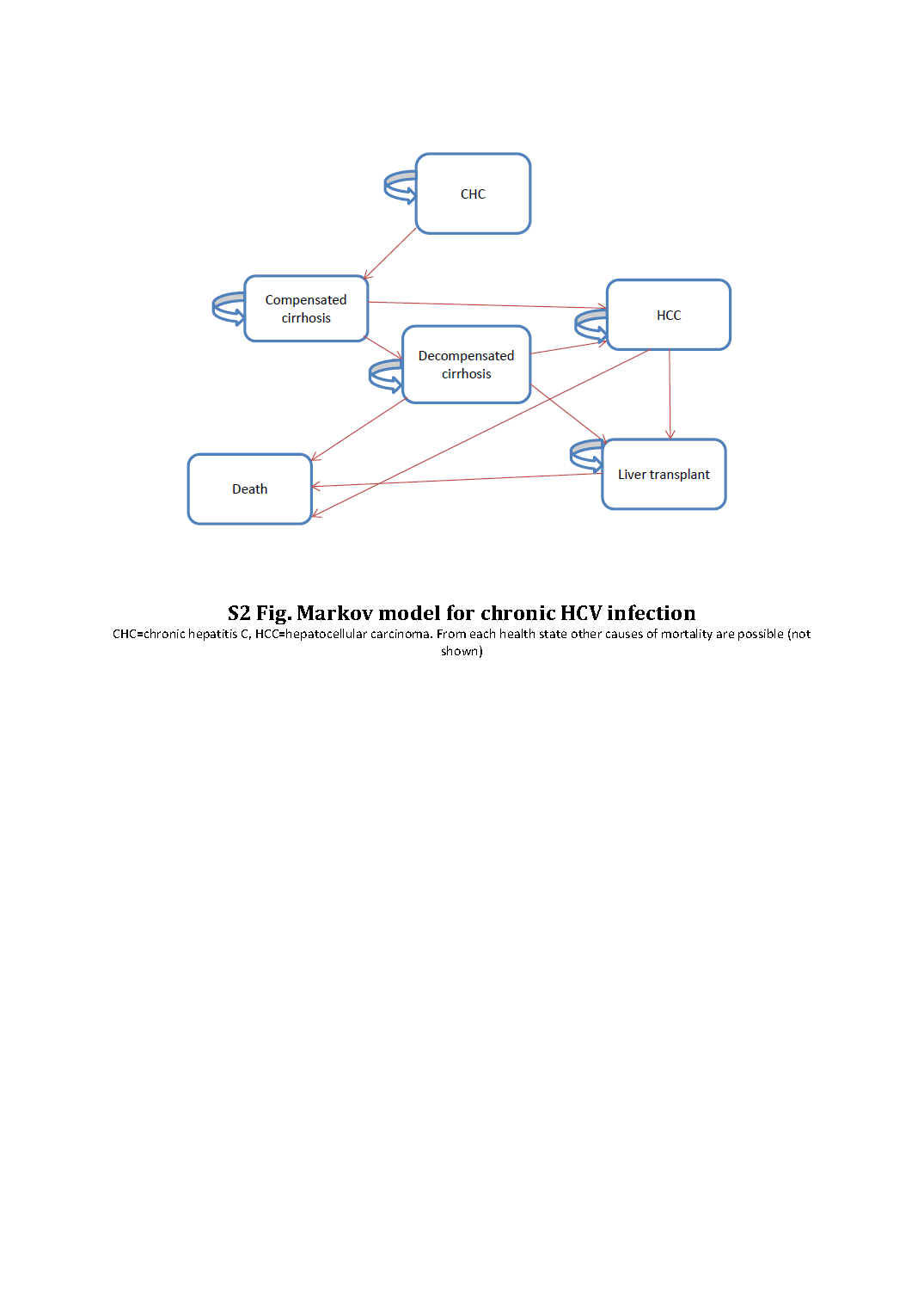

Supplement: S2 Fig — (TIFF) [file pone.0207037.s009.tiff]
